# Supplementary material for: A searchable atlas of pathogen-sensitive lncRNA networks in human macrophages
Source: Nat Commun. 2025 May 21;16:4733. doi: 10.1038/s41467-025-60084-x (PMC12095776; doi:10.1038/s41467-025-60084-x)
Supplement: Supplementary file 1 — Supplementary Information [file 41467_2025_60084_MOESM1_ESM.pdf]

SUPPLEMENTARY INFORMATION

A searchable atlas of pathogen-sensitive lncRNA networks in human macrophages

Nils Schmerer, Harshavardhan Janga, Michelle Aillaud, Janina Hoffmann, Marina Aznaourova, Sarah Wende, Henrike Steding, Luke D. Halder, Michael Uhl, Fabian Boldt, Thorsten Stiewe, Andrea Nist, Lukas Jerrentrup, Andreas Kirschbaum, Clemens Ruppert, Oliver Rossbach, Evgenia Ntini, Annalisa Marsico, Chanil Valasarajan, Rolf Backofen, Uwe Linne, Soni S. Pullamsetti, Bernd Schmeck, Leon N Schulte*

*Corresponding author.

Email: leon.schulte@uni-marburg.de

CONTENT	PAGE

SUPPLEMENTARY FIGURES	3-19
SUPPLEMENTARY TABLES	20-27
SUPPLEMENTARY NOTE 1	28-40

SUPPLEMENTARY FIGURES

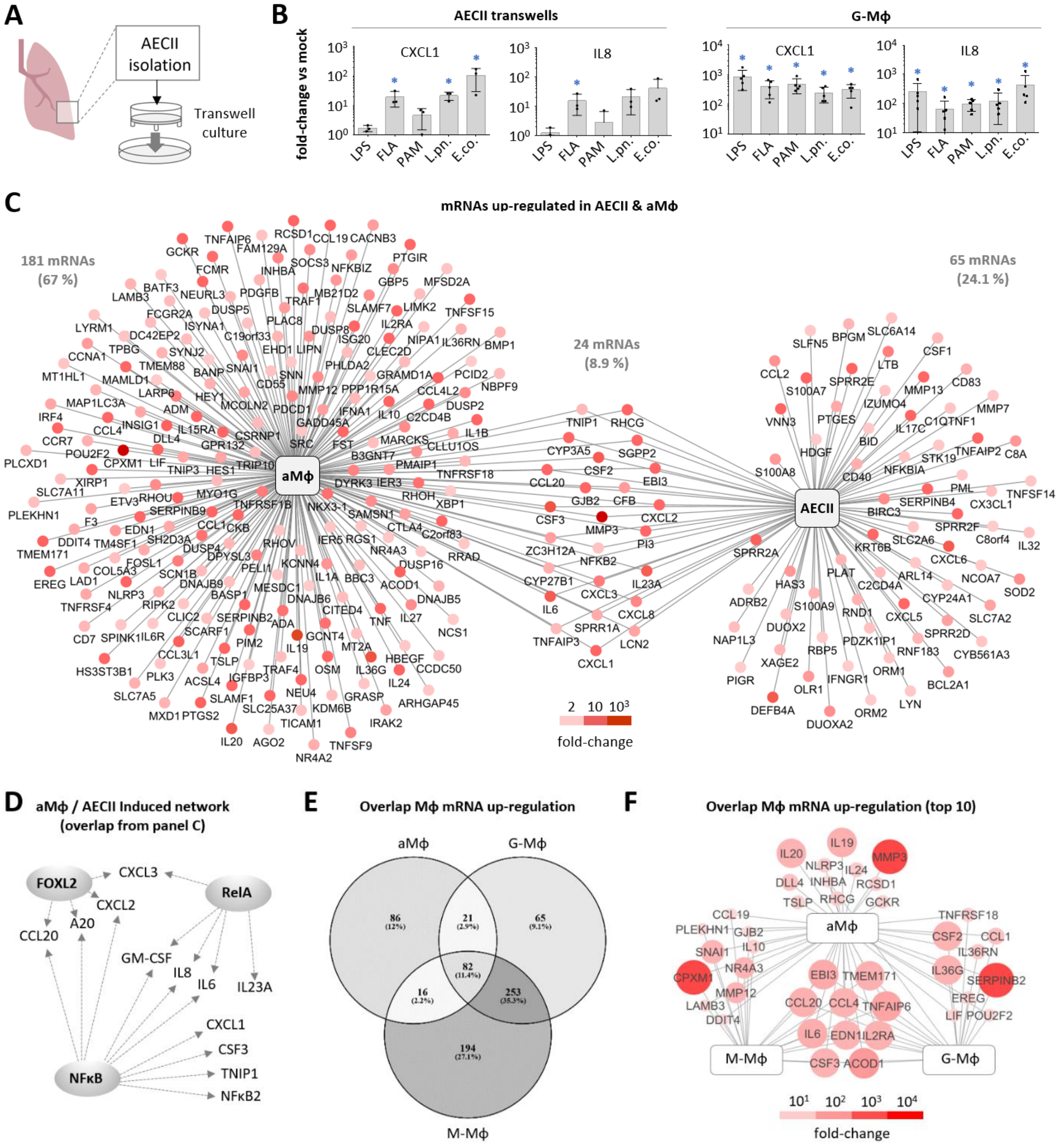

Supplementary Figure 1: Comparison of alveolar macrophage and epithelial immune responses. A) Illustration of AECII cell isolation and cultivation procedure. **B)** RT-qPCR analysis of inflammatory gene induction in AECII cells (three independent experiments) and G-MΦ (five independent experiments) in response to 4 h stimulation with TLR4-ligand LPS, TLR5-ligand flagellin (FLA), TLR2-ligand Pam3CSK4 (PAM), *Legionella pneumophila* (L.pn.) and *Escherichia coli* (E. co.). Mean values +/- SD. One-way ANOVA tests were conducted (relative to mock control). Statistical significance ($p \leq 0.05$) is indicated by asterisks. Exact p-values in the same order (left to right) as the asterisks: AECII (CXCL1): 0.024, 0.011, 0.048; AECII (IL8): 0.048; G-MΦ (CXCL1): <0.001, <0.001, <0.001, <0.001, <0.001; G-MΦ (IL8): <0.001, 0.003, <0.001, <0.001, <0.001. **C)** Cytoscape networks of mRNAs up-regulated ≥ 2-fold in both replicates of 4 h LPS-stimulation (aMΦ) and FLA-stimulation (AECII) RNA-seq experiments. Fold-change color-coded. **D)** ConsensusPathDB-predicted transcription factor network driving expression of genes commonly induced in aMΦ and AECII (overlap from C). **E)** Venn diagram showing shared and unique mRNA regulations ≥ 2-fold, recorded in aMΦ, G-MΦ and M-MΦ (RNA-seq experiments from Fig. 1C-D). **F)** Cytoscape

network aggregating the top10 induced mRNAs in aMΦ, G-MΦ and M-MΦ, respectively (RNA-seq experiments from Fig. 1C-D).

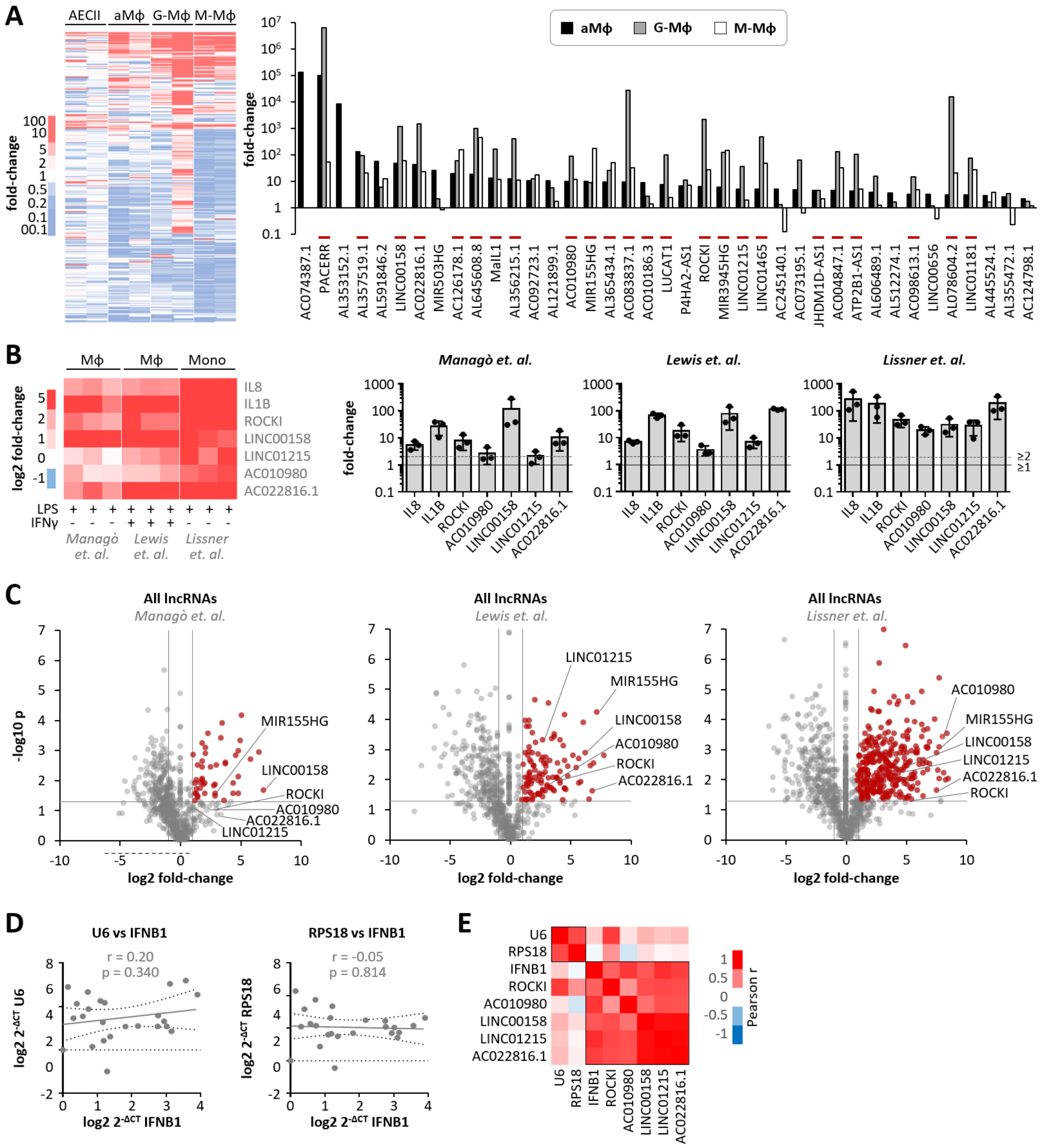

Supplementary Figure 2: Analysis of lncRNA gene-regulation in macrophages. A) Left: Same as Fig. 1E, but showing lncRNAs regulated ≥ 2-fold (up or down) in either AECII, aMΦ, G-MΦ or M-MΦ in both RNA-seq replicates. Right: Bar-plot showing all lncRNAs up-regulated ≥ 2-fold in response to LPS in both replicates of the aMΦ experiment (mean fold-changes shown). Regulations in the G-MΦ and M-MΦ datasets shown for comparison. LncRNAs regulated ≥ 2-fold in all replicates of both the aMΦ and the G-MΦ experiment (Fig. 1E-F analysis) are marked with red horizontal bars. **B)** Re-analysis of published RNA-seq data, showing the regulation of selected lncRNAs in blood-derived human macrophages and monocytes (Mono) stimulated with LPS and IFNγ (6 h) as indicated (three RNA-seq replicates per dataset). Left: color-coded heatmap. Right: bar plot representation (mean +/- SD), with 1- and 2-fold cutoff lines. **C)** Volcano plot representation of lncRNA regulations in response to LPS (or LPS + IFNγ for Lewis at al.) in the RNA-seq datasets analyzed in B). Significantly up-regulated lncRNAs (fold-change ≥ 2, p-value ≤ 0.05 [two-tailed Student's t-test]) are highlighted in red. LncRNAs in the focus of this study and MIR155HG as a control for successful immune-activation are indicated. **D)** Linear regression plots with 95% confidence intervals (dashed lines) and Pearson correlation statistics (two-tailed test), comparing the RT-qPCR-determined levels of U6 snRNA and RPS18 mRNA with IFNB1 (log2 $2^{-\Delta CT}$ values) in

human bronchoalveolar lavage cell pellets. **E)** Pearson correlation matrix comparing the five lncRNAs, IFNB1, U6 snRNA and RPS18 mRNA across the patient cohort data shown in Fig. 1I and Supplementary Fig. 2D.

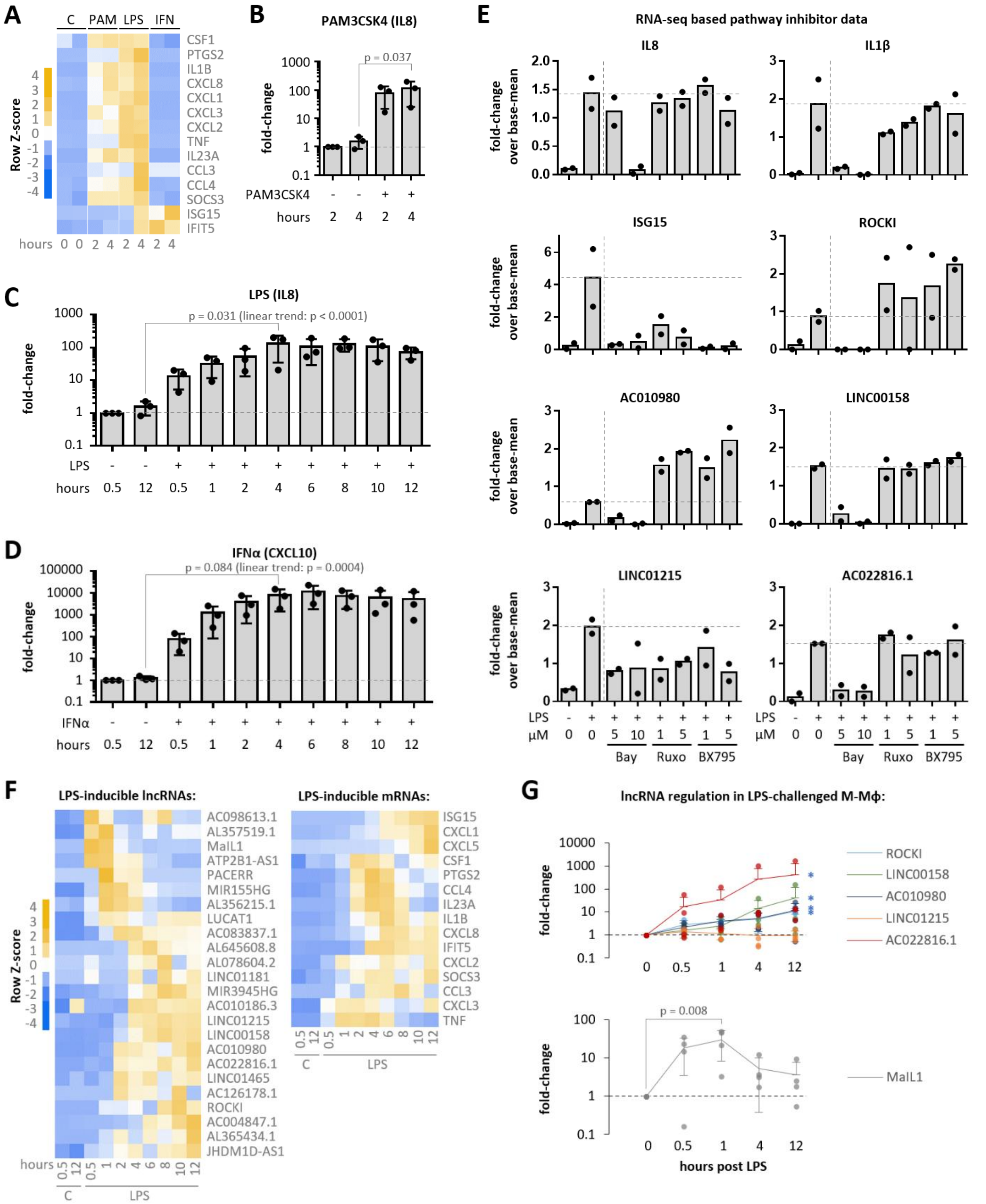

Supplementary Figure 3: Pathway dependency and temporal regulation of lncRNAs and mRNAs in G-MΦ and M-MΦ. A) Same as Fig. 2B, but for selected immune-marker mRNAs. **B-D)** RT-qPCR validation of immune marker gene induction in response to the indicated immune stimuli (G-MΦ, various stimulation time-points; three independent experiments; mean values +/- SD). **E)** Bar plots summarizing the regulation of key immune marker genes and lncRNAs in response to LPS and pathway inhibitors (RNA-seq analysis, using samples from two experimental replicates of the experiment shown in Fig. 2C). **F)** Left: Z-score heatmap showing the expression of LPS-inducible lncRNAs (fold-change ≥ 2 in all aMΦ and G-MΦ replicates from Supplementary Fig. 2A) at various time-points post stimulation (RNA-seq analysis using samples from two experimental replicates of the experiment shown in panel C. Replicate RPKMs were averaged for Z-score calculation). Right: same as left panel, but showing key immune-responsive mRNAs. **G)** Time-series RT-qPCR analysis of lncRNA expression in LPS-

treated M-MΦ (four independent experiments; mean values +/- SD). Exact p-values: ROCKI: <0.001, LINC00158: 0.026, AC010980: 0.002, AC022816.1: 0.020. **B,C,D,G**: One-way ANOVA tests were conducted. In C, D, and G, p-values were calculated for the linear trends of column averages from left to right or alternatively for the indicated comparisons. Where applicable, exact p-values are shown; otherwise, statistical significance ($p \leq 0.05$) is indicated by asterisks.

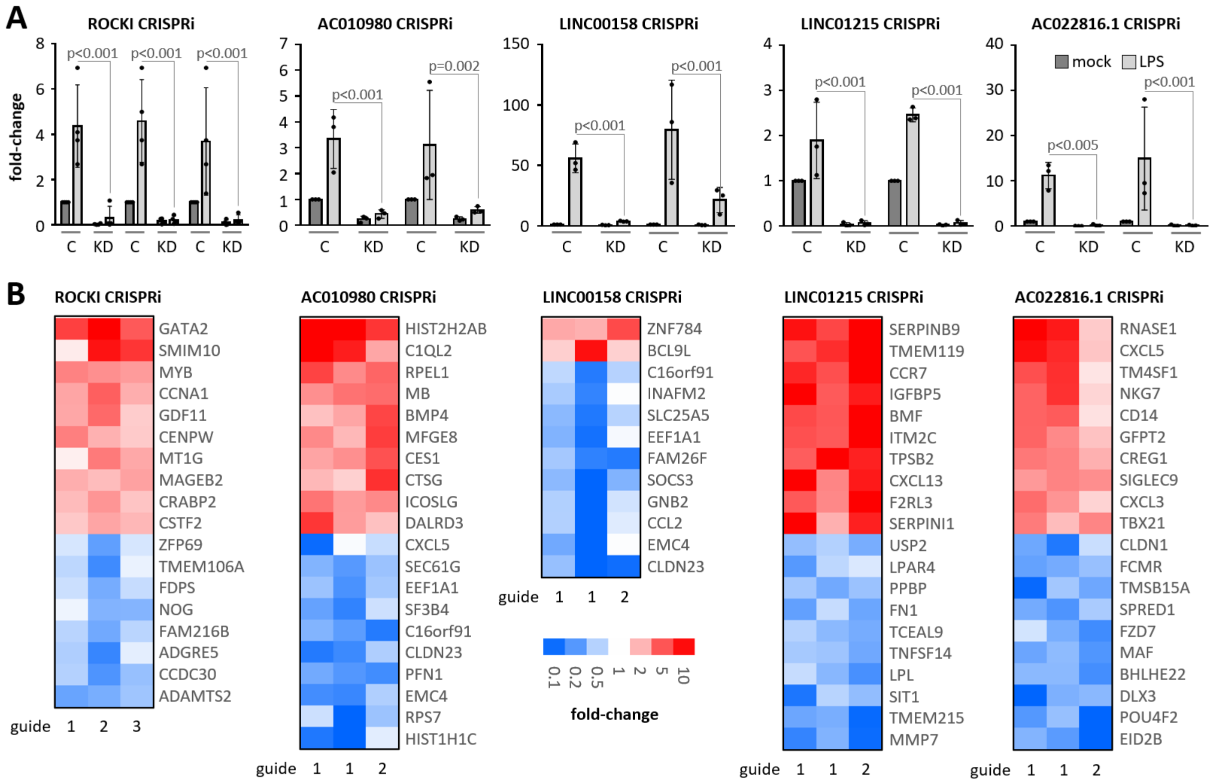

Supplementary Figure 4: Silencing of LPS-inducible lncRNAs in THP1 cells using CRISPRi. A) RT-qPCR analysis, showing the successful CRISPRi-based knockdown of lncRNAs in 8 h mock and LPS treated THP1 cells. Each knockdown represents a different guideRNA used for CRISPRi. ≥ three independent experiments per guideRNA; mean-values +/- SD. One-way ANOVA tests were conducted and exact p-values are shown for the indicated comparisons. **B)** Heatmaps showing the top 10 mRNAs significantly up-regulated and down-regulated ≥ 2-fold ($p \leq 0.05$) in response to silencing of the indicated lncRNAs (RNA-seq analysis with 3 independent replicates using samples introduced in panel A). If less than 10 mRNAs are shown, not enough mRNAs passed the fold-change and significance cutoffs described above.

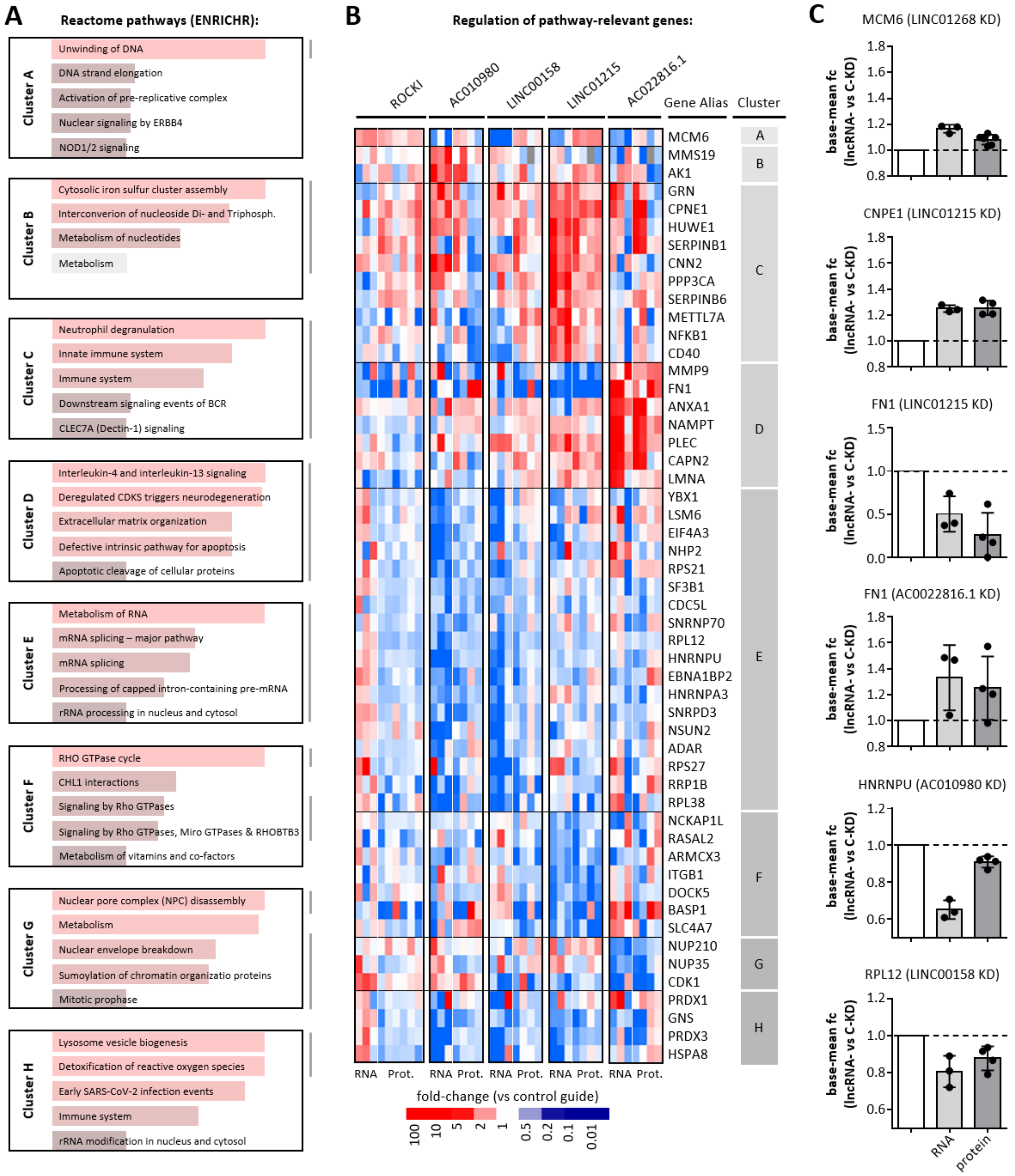

Supplementary Figure 5: Pathway analysis of factors regulated in response to lncRNA silencing. A) Top5 Reactome pathways associated with the factors involved in the clusters from Fig. 4B. **B)** Same as Fig. 4B, but reduced to factors included in the Reactome pathways shown in panel A. **C)** Bar plots showing the regulation of selected pathway-relevant factors from B at the RNA and protein level upon silencing of the lncRNA they respond to most strongly (≥ three independent experiments; mean +/- SD).

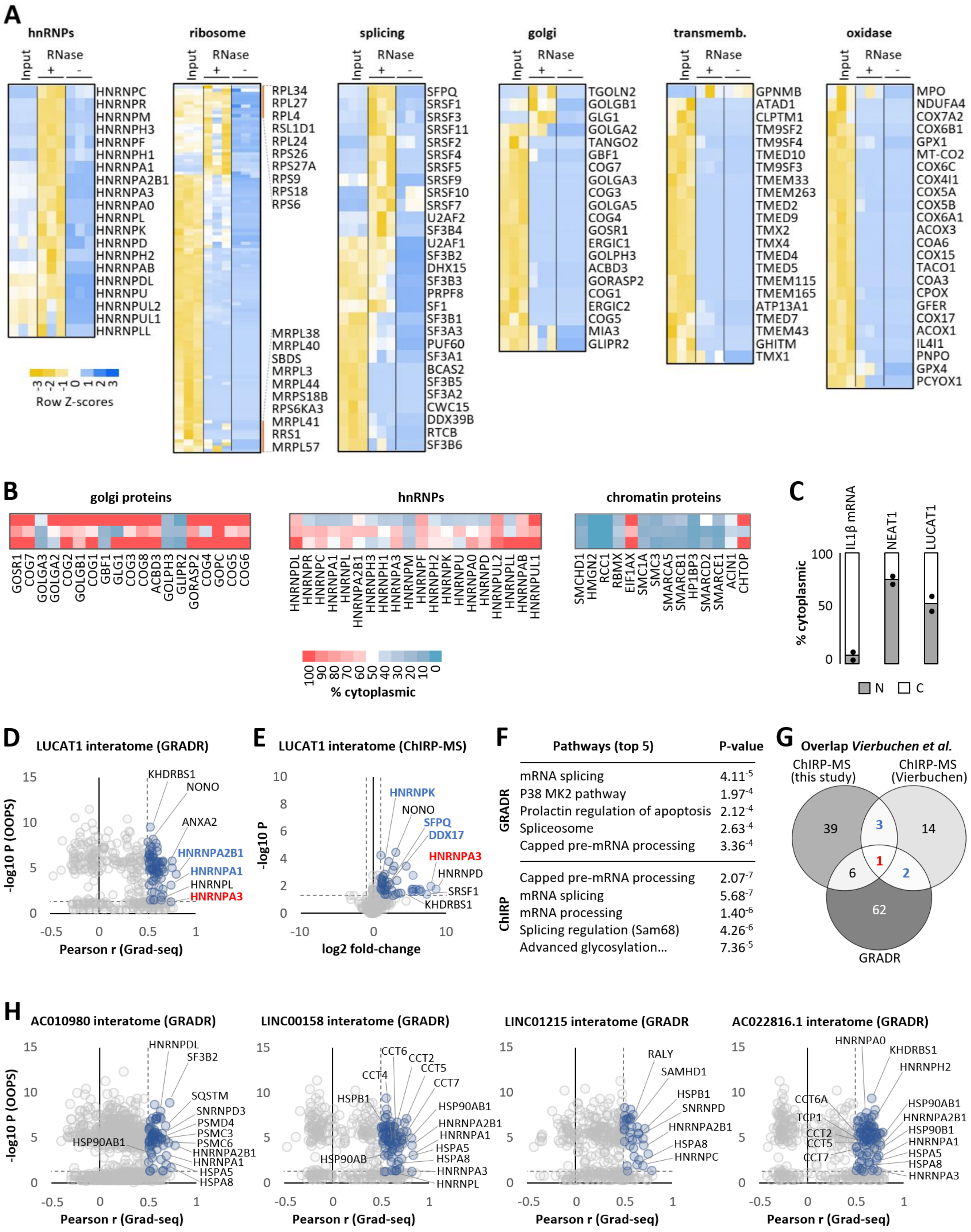

Supplementary Figure 6: Establishment of GRADR for lncRNA-protein interactome studies. A) Row Z-score heatmaps showing the abundance of different protein classes in input, RNase-based elution and control elution (no RNase) fractions of three independent OOPS-MS experiments (data from Fig. 5B). **B)** Heatmaps showing the percentages of cytoplasmic localization for different protein classes, shown in Fig. 5C. **C)** Subcellular fractionation controls for experiment shown in Fig. 5D and F fractions and LUCAT1 subcellular localization (RNA-seq, averages and standard deviation from two independent experiments). **D)** LUCAT1 GRADR interactome plot (analogous to Fig. 5E). LUCAT1 interactors also identified by Vierbuchen et al. are highlighted (blue: identified in panel D + in Vierbuchen et al.; red: identified in panel D, Vierbuchen et al. and in panel E). **E)** Volcano plot highlighting ChIRP-MS identified LUCAT1 interactors in LPS-stimulated G-MΦ. Interactors also identified by Vierbuchen et al. and

GRADR are highlighted analogous to panel D. **F)** Top 5 Bioplanet pathways associated with the LUCAT1 interactors predicted by GRADR ($r \geq 0.5$, $p \leq 0.05$) and ChIRP-MS (fold-change ≥ 2, $p \leq 0.05$), respectively. **G)** Venn diagram showing the overlap of our GRADR- and ChIRP-MS-based LUCAT1 interactor predictions and the ChIRP-MS-based predictions from Vierbuchen et al. **H)** Individual GRADR interactome plots for the lncRNAs AC010980, LINC00158, LINC01215 and AC022816.1 (analogous to Fig. 5E). **D, E, H)**: P-values calculated using a two-tailed Student's t-test.

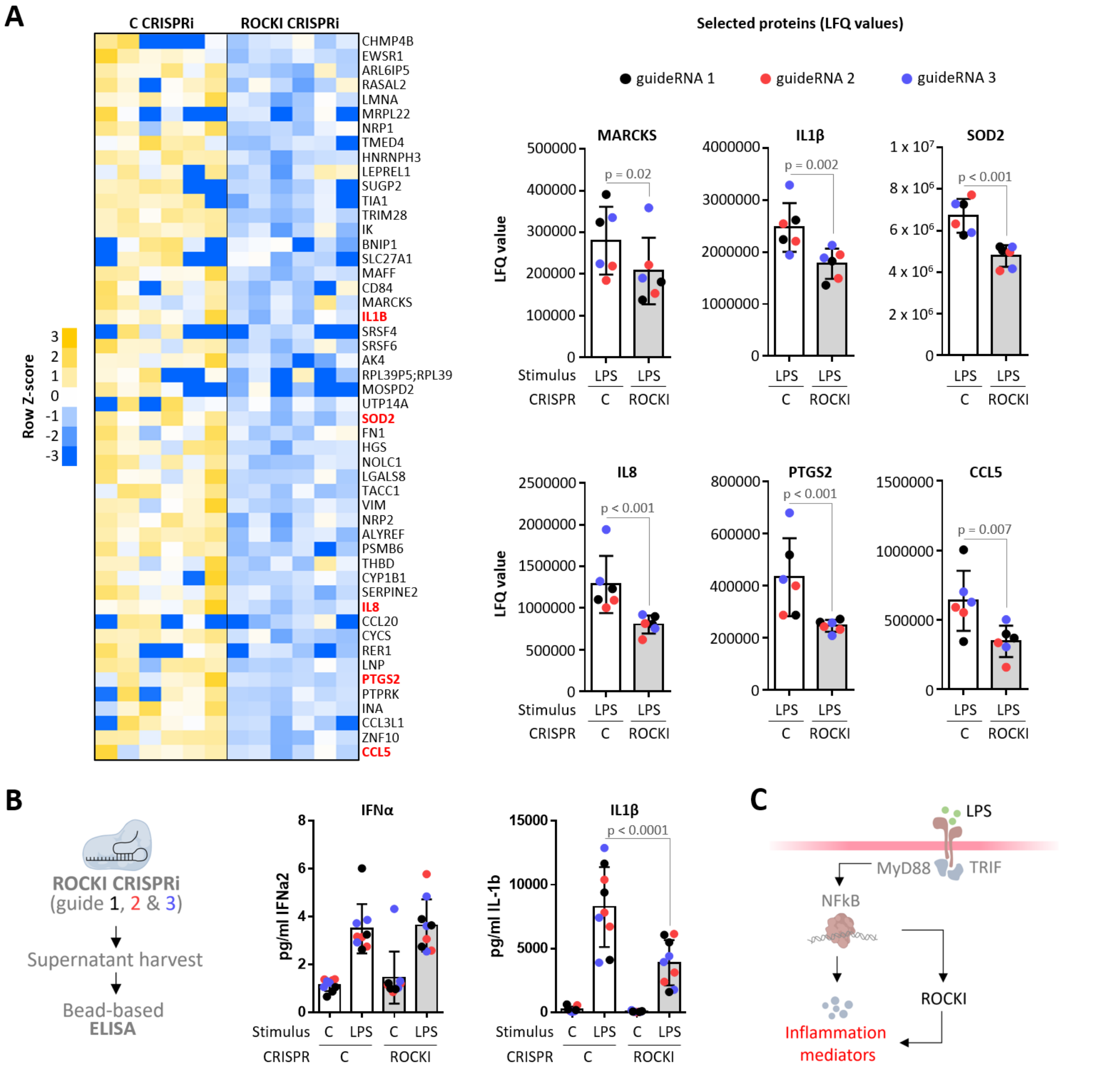

Supplementary Figure 7: Impact of ROCKI on protein expression. A) Left: Row Z-score heatmap showing proteins significantly down-regulated (≥ 2-fold down, $p \leq 0.05$) upon ROCKI silencing (experiment from Fig. 4B). Key immune response genes are highlighted in red. Right: LFQ abundances of selected immune-related proteins in ROCKI silenced and control THP1 cells (6 independent experiments; mean +/- SD). Experiments corresponding to the different guideRNAs used are highlighted with different colors. Significant differences are indicated (two-tailed Student's t-test p-values). **B)** Left: Illustration of ELISA experiment. Middle and Right: IFNα and IL1β ELISA results with supernatants from the control and ROCKI silenced cells, also used in panel A (9 independent experiments; mean +/- SD). Significant differences are indicated (One-way ANOVA test p-values). **C)** Model of ROCKI function as a positive regulator of inflammatory mediator production in TLR-triggered immunity. All panels: 8h LPS stimulated THP1 cells.

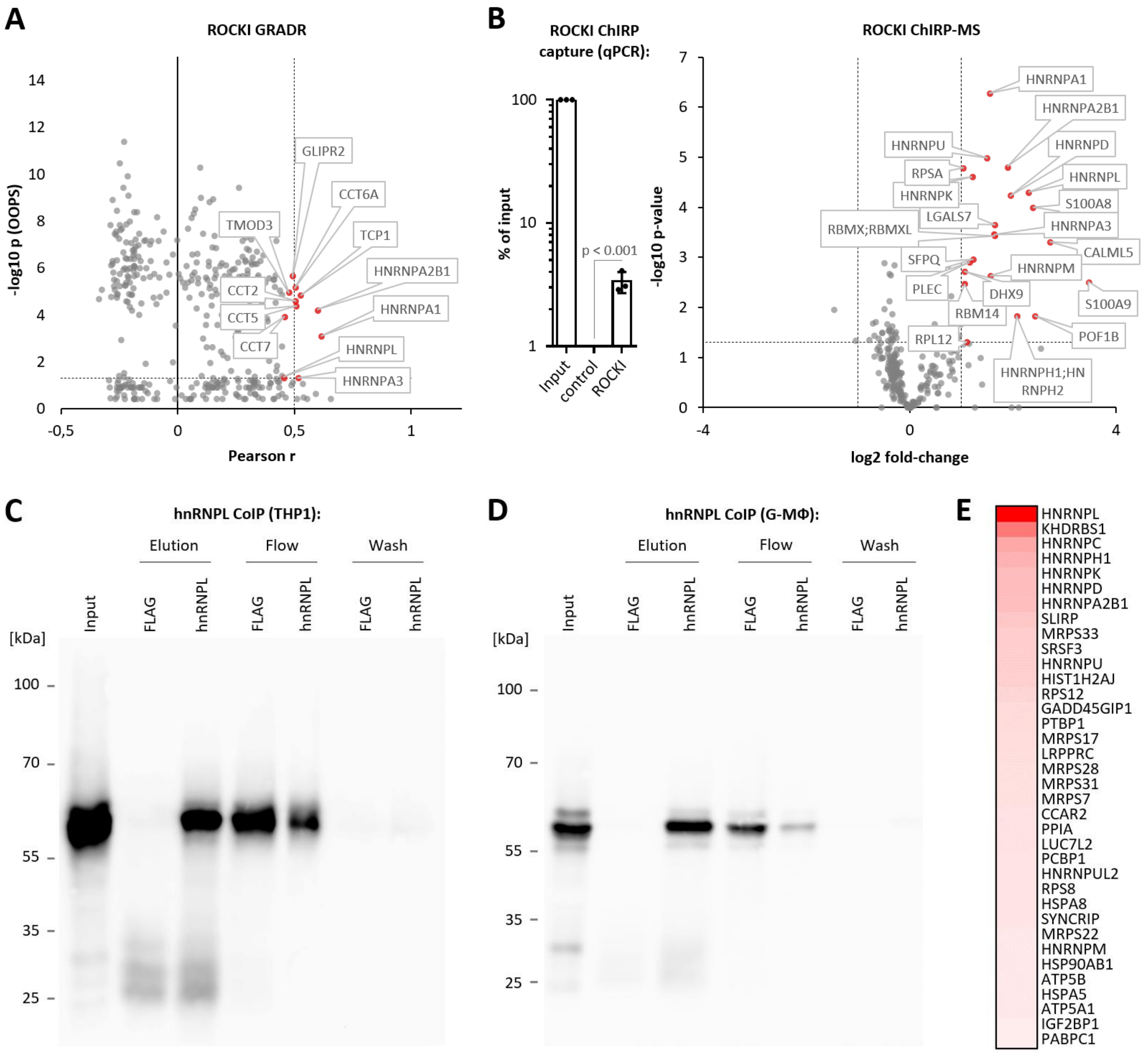

Supplementary Figure 8: Analysis of ROCKI-hnRNP interaction by GRADR, ChIRP-MS and UV-CLIP. A) Magnified view of ROCKI interactome prediction by GRADR, shown in Fig. 6A (p-values based on two-tailed Student's t-test). **B)** Left: RT-qPCR-confirmation of ROCKI pull-down for ChIRP-MS (relative RNA abundance in control ChIRP eluate and ROCKI ChIRP eluate compared to input cell lysate; three independent experiments; mean values +/- SD). One-way ANOVA was used to determine statistical significances (p-value indicated). Right: Magnified view of ROCKI interactome prediction by ChIRP-MS, shown in Fig. 6C. 8 h LPS stimulated THP1 cells. P-values calculated using a two-tailed Student's t-test. **C-D)** Representative Western Blot full scans from three independent hnRNP L CLIP experiments using 8 h LPS-stimulated THP1 cells and 4 h LPS-stimulated G-MΦ, shown in Fig. 6D. **E)** Complete view of hnRNP L co-eluted proteins (magnification of heatmap from Fig. 6E).

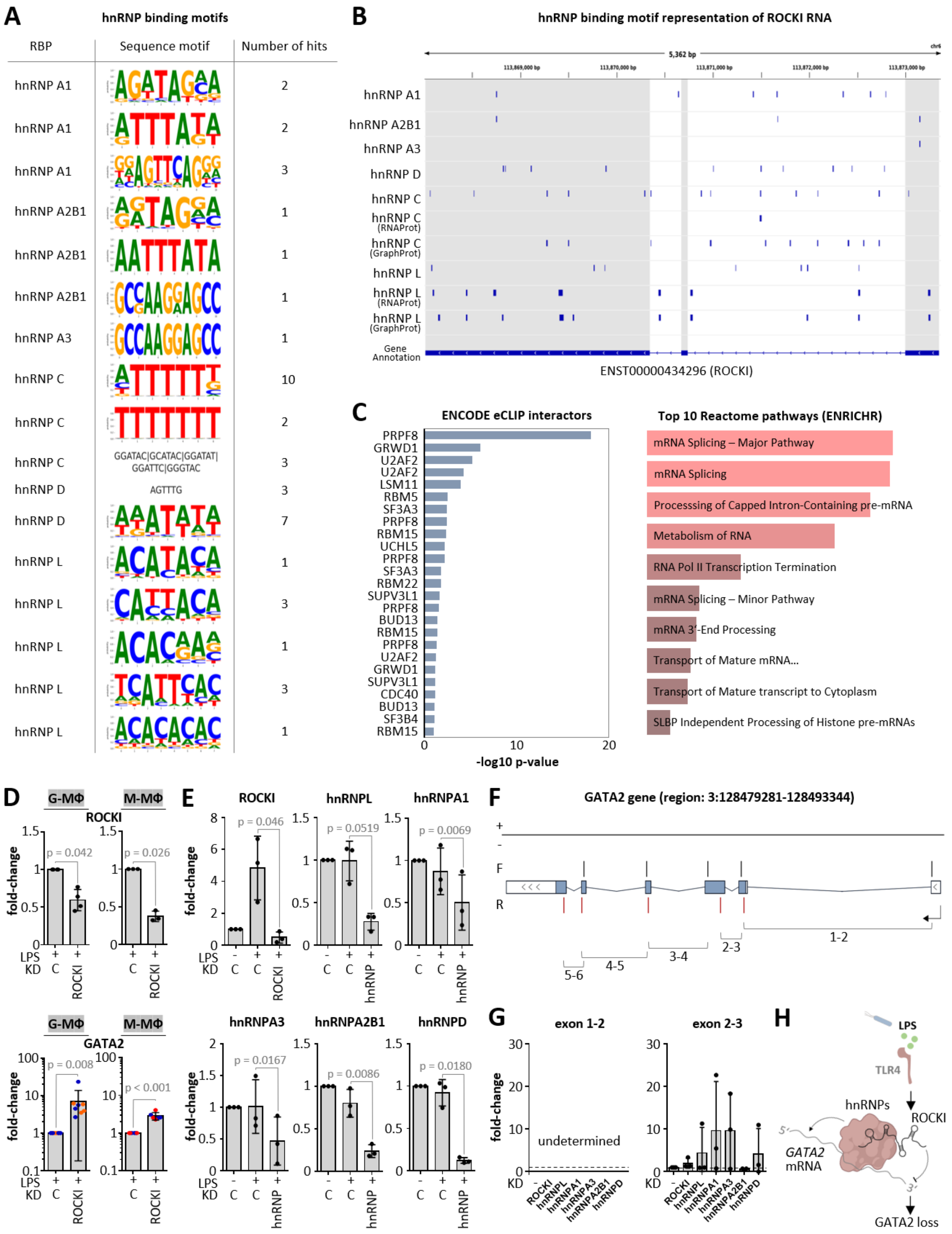

Supplementary Figure 9: Substantiation of ROCKI-hnRNP dependent roles in mRNA processing. A) Sequence motifs recognized by hnRNP proteins extracted from the literature with associated motif hit numbers in the ROCKI transcript ENST00000434296. For the motif search, sequence motifs in MEME format (visualized as sequence logos) or regular expressions / strings (for hnRNP C and hnRNP D) were used. Sequence motifs were extracted from catRAPID omics v2.0 and ATtRACT curated motif databases (see methods). **B)** ROCKI genomic region (IGV screenshot) with tracks containing sequence motif hits and predicted binding sites for hnRNP proteins from panel A and binding sites of HNRPN C and HNRNP L predicted by two computational methods (GraphProt and RNAProt). Gray = ROCKI (ENST00000434296) exon regions. **C)** Left panel: ENCODE eCLIP data based

prediction of ROCKI interacting proteins. Right panel: Reactome pathway analysis with proteins from right panel (top 10 pathways shown). **D)** RT-qPCR validation of ROCKI knockdown (KD) and *GATA2* mRNA regulation in either control (C) or ROCKI siRNA transfected, LPS-stimulated G-MΦ or M-MΦ. Lower panel: RT-qPCRs with primers spanning the *GATA2* exon 4-5 (red) and 5-6 (blue) junctions were conducted. ≥ three independent experiments; mean values +/- SD. Statistical significances (two-tailed Student's t-test p-values) are shown. **E)** RT-qPCR validation of ROCKI, hnRNP L, hnRNP A1, hnRNP A3, hnRNP A2B1 and hnRNP D knockdown (KD) in 8 h LPS-treated THP1 cells, respectively. C = control knockdown. Three independent experiments; mean values +/- SD. One-way ANOVA test p-values are shown. **F)** Illustration of the GATA2 mRNA exon architecture (based on *ENSEMBL*/*MANE*-transcript GATA2-201), with forward (F) and reverse (R) primer locations and used combinations indicated. + and - denote the sense and antisense DNA strand. **G)** RT-qPCR analysis of GATA2 mRNA levels using primers targeting exon-exon junctions 1-2 and 2-3 in THP1 cells upon indicated ROCKI CRISPRi-knockdown and hnRNP GapmeR-knockdown (KD), respectively. - = control-CRISPRi or -GapmeR knockdown. 8 h LPS-treated THP1 cells. Three independent experiments; mean values +/- SD. No significant changes compared to control KD detected (One-way ANOVA test). **H)** Model of ROCKI-interaction with hnRNPs and GATA2 mRNA suppression during macrophage immune-activation.

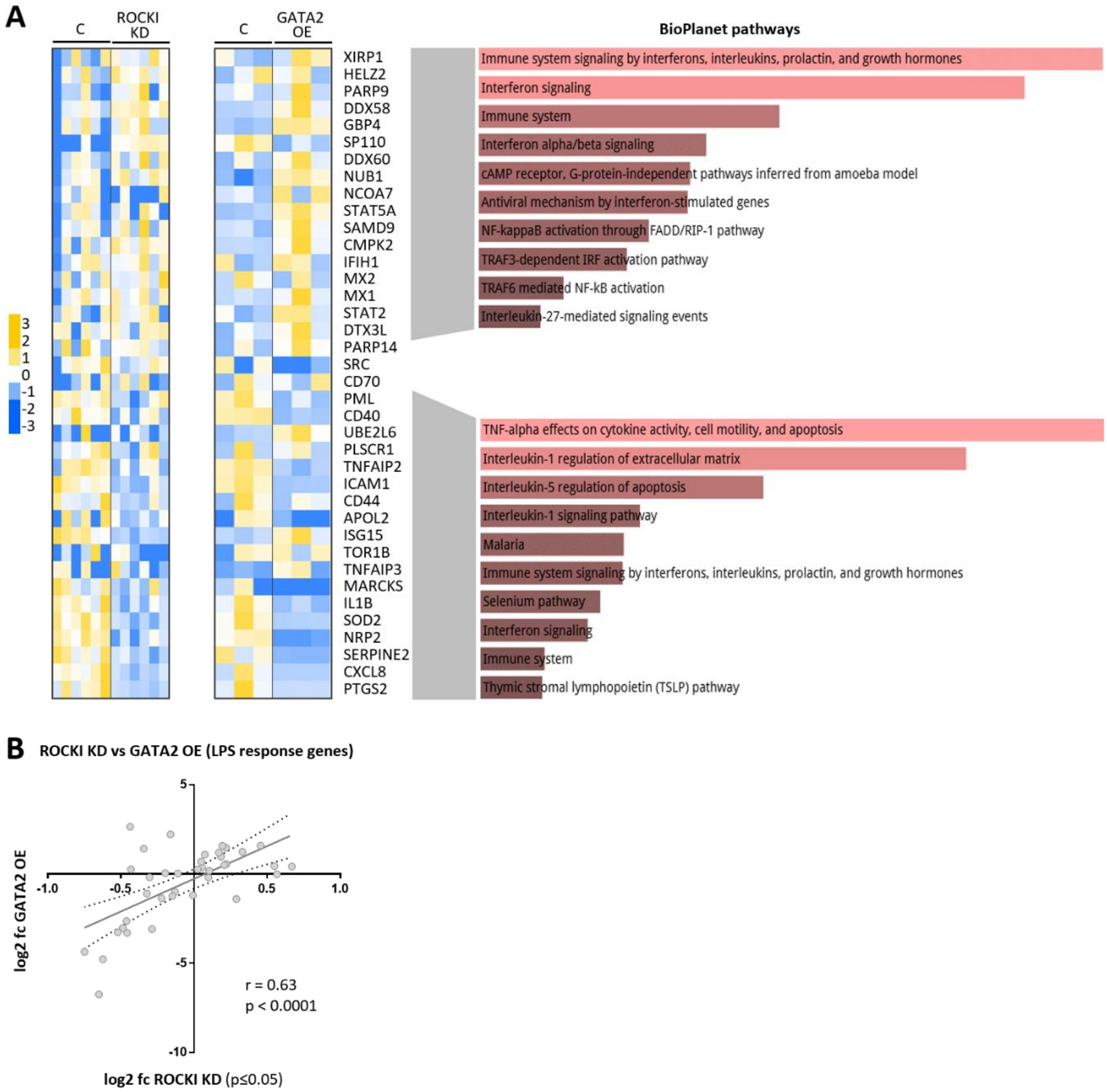

Supplementary Figure 10: Comparison of the influence of ROCKI and GATA2 on protein expression. A) Left: Clustered Z-score heatmaps showing proteins detected in both the ROCKI knockdown and GATA2 overexpression proteomics datasets and regulated (≥ 2-fold up or down, p ≤ 0.05, two-tailed Student's t-test) in one of the two datasets (experiments from Fig. 4B and Fig. 6I; 8 h LPS-stimulated THP1 cells). Right: Bioplanet pathway predictions for the indicated gene sets. **B)** Scatter plot comparing the regulation of LPS-responsive (≥ 2-fold up or down, p ≤ 0.05, two-tailed Student's t-test) proteins upon ROCKI knockdown and GATA2 overexpression (same as Fig. 6I, but reduced to proteins marked as LPS-responsive in Fig. 4D dataset). Pearson correlation analysis statistics are shown (two-tailed test).

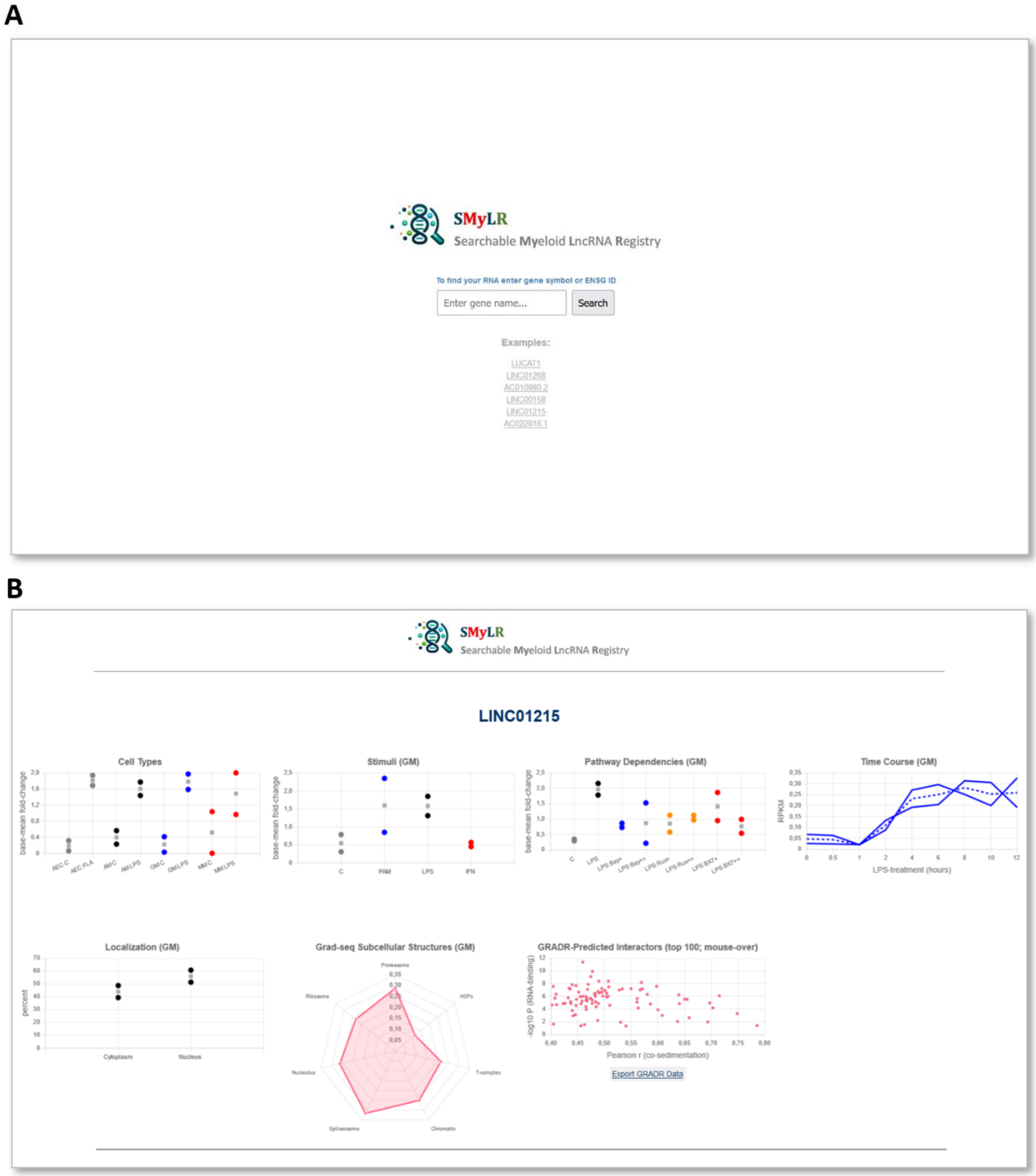

Supplementary Figure 11: Screenshots of the SMyLR web page. A) SMyLR start page. **B)** SMyLR results page (in this example *LINC01215* was queried).

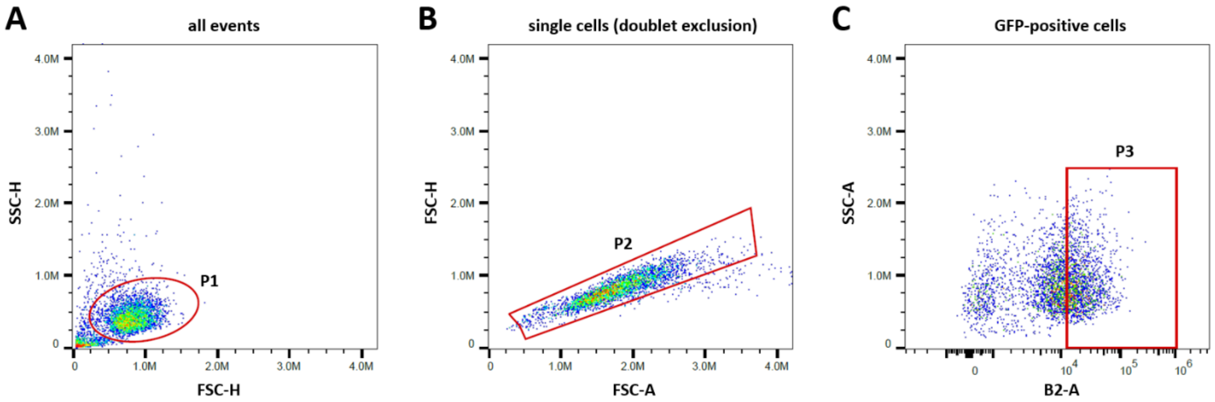

Supplementary Figure 12: Cell sorting strategy for enrichment of CRISPRi vector transduced THP1 cells. A) Representation of control CRISPRi vector transduced THP1 cells in the FSC-H vs SSC-H channel, and discrimination of cells ("P1" gate) from debris. **B)** Representation of cells selected in panel A in the FSC-A vs FSC-H channel and gating for doublet exclusion ("P2" gate). **C)** Representation of cells gated in panel B in the B2-A (GFP) vs SSC-A channel and selection of cells expressing the *GFP* reporter gene from the CRISPRi vector ("P3" gate).

SUPPLEMENTARY TABLES

Supplementary Table 1: Patients undergoing bronchoalveolar lavage (ranked by IFNB1 levels [Fig. 1I]).

**IFNB1 log2 $2^{-\Delta CT}$**	**Anonymized patient ID**	**Sex**	**Age (years)**	**Indication / Disease**
3.905	14	female	62	Dystelectasis
3.569	13	female	45	Lymphadenopathy
3.150	10	female	38	Interstitial lung disease
3.107	11	male	66	Idiopathic coughing
3.014	24	male	78	Interstitial lung disease
2.941	12	female	68	Interstitial lung disease
2.766	23	female	61	Interstitial lung disease
2.752	22	male	78	Interstitial lung disease
2.179	21	female	61	Chronic bronchitis
1.818	1	male	69	Lymphadenopathy
1.374	15	female	55	Lymphadenopathy
1.286	20	male	50	Lymphadenopathy
1.215	16	male	68	Interstitial lung disease
1.185	9	male	80	Interstitial lung disease
1.161	19	female	31	Lymphadenopathy
1.094	8	male	82	Atypical pneumonia
0.855	2	female	66	Interstitial lung disease
0.744	17	female	58	Interstitial lung disease
0.726	18	female	61	Interstitial lung disease
0.578	7	male	60	Interstitial lung disease
0.405	6	male	32	Lymphadenopathy
0.306	4	male	67	Lymphadenopathy
0.148	5	female	71	Interstitial lung disease
0.000	3	male	77	Lymphadenopathy

Supplementary Table 2: gRNAs used in the present study. Underlined sequences represent the cloning overhangs.

gRNA description	DNA oligo internal ID	DNA oligo sequence
ROCKI (gRNA insert 1)	OBS-2693	CACCGTCTATACTCACCGCCATCTC
	OBS-2694	AAACGAGATGGCGGTGAGTATAGAC
ROCKI (gRNA insert 2)	OBS-2695	CACCGGTCTCTTGAACTTCGGACCT
	OBS-2696	AAACAGGTCCGAAGTTCAAGAGACC
ROCKI (gRNA insert 3)	OBS-2697	CACCGTAAAGCACCACAACGCATGT
	OBS-2698	AAACACATGCGTTGTGGTGCTTTAC
LINC00158 (gRNA insert 1)	OBS-3141	CACCGCAGACACATCTATAGTTAAG
	OBS-3142	AAACCTTAACTATAGATGTGTCTGC
LINC00158 (gRNA insert 2)	OBS-3143	CACCGATTGAACATACCTTTTATCG
	OBS-3144	AAACCGATAAAAGGTATGTTCAATC
LINC01215 (gRNA insert 1)	OBS-2325	CACCGCTTACAGATGGCTGTTGATA
	OBS-2326	AAACTATCAACAGCCATCTGTAAGC
LINC01215 (gRNA insert 2)	OBS-2327	CACCGTTCTCAGGAGATCTGATAAG
	OBS-2328	AAACCTTATCAGATCTCCTGAGAAC
AC010980.1 (gRNA insert 1)	OBS-2735	CACCGGCTTCCTAGCGGTCCTGGAA
	OBS-2736	AAACTTCCAGGACCGCTAGGAAGCC
AC010980.1 (gRNA insert 2)	OBS-2737	CACCGAATGGACTTGAATTGCCCGG
	OBS-2738	AAACCCGGGCAATTCAAGTCCATTC
AC022816.1 (gRNA insert 1)	OBS-2747	CACCGCTGTTGCAAGCTATTGGTGG
	OBS-2748	AAACCCACCAATAGCTTGCAACAGC
AC022816.1 (gRNA insert 2)	OBS-3682	CACCGACTGAGCCTAGTTGGTCACA
	OBS-3683	AAACTGTGACCAACTAGGCTCAGTC

Supplementary Table 3: Antisense LNA GapmeRs and siRNAs. "*" = Phosphorothioate backbone modifications.

**Description**	**Source**	**GapmeR or siRNA sequence**	**Identifier**
Antisense LNA GapmeR control	Qiagen	A*A*C*A*C*G*T*C*T*A*T*A*C*G*C	Negative control A, 339515 LG00000002-DDA
Antisense LNA GapmeR hnRNP L	Qiagen	G*T*G*A*T*C*G*A*A*T*A*A*A*T*G*G	HNRNPL_1, 339511 LG00844161-DDA
Antisense LNA GapmeR hnRNP A1	Qiagen	G*C*T*T*A*A*G*A*G*T*A*G*A*C*T*A	HNRNPA1_2, 339511 LG00844142-DDA
Antisense LNA GapmeR hnRNP A3	Qiagen	A*C*A*C*G*C*G*T*A*G*A*A*A*T*T*G	HNRNPA3-201_1, 339511 LG00844591-DDA
Antisense LNA GapmeR hnRNP A2B1	Qiagen	G*A*C*C*G*T*A*G*T*T*A*G*A*A*G*G	HNRNPA2B1_1, 339551 LG00844131-DDA
Antisense LNA GapmeR hnRNP D	Qiagen	A*G*T*A*G*T*C*C*T*T*C*A*G*A*T*C	HNRNPD_201_3, 339511 LG00844623-DDA
Control siRNA	Invitrogen	undisclosed	4390846
ROCKI siRNA	Invitrogen	AGUACAUGGAGUAACGUGUtt	-

Supplementary Table 4: ChIRP-seq and ChIRP-MS oligonucleotides used in the present study.

Probe target	DNA oligo internal ID	DNA oligo sequence
ROCKI	OBS-2909	CGGATAGTCAGACTTCTCAC
ROCKI	OBS-2910	ATTGGATGTTTGCTGGGATG
ROCKI	OBS-2911	TCAAGATCATGACCGTATCC
ROCKI	OBS-2912	CTTTGGCAGGATTACTTCAC
ROCKI	OBS-2913	TGATGCTCAGTTACTGTTTT
ROCKI	OBS-2914	GATTGTACCTCATGCTGTAG
ROCKI	OBS-2915	GGTTAAGGGATAGCCAGATA
ROCKI	OBS-2916	CCACCCAAATCTCATCTTGA
LUCAT1	OBS-3186	GAGGATGAAAGCTGTTCTTA
LUCAT1	OBS-3187	ATCCGAGCTTGACACATGGT
LUCAT1	OBS-3188	GGTCTCTGGTGCCAAGGTCC
LUCAT1	OBS-3189	GAGCTTCTTGTGAGGAAAGG
LUCAT1	OBS-3190	CGTGAGAGAAATACAAGAAA
LUCAT1	OBS-3191	GTTGCTGTTAGAAAACTTCA
LUCAT1	OBS-3192	TTGTGAGGGGATGAGAATAC
LUCAT1	OBS-3193	TTAGTGACTGAAATGTGTGA
LUCAT1	OBS-3194	CTTCCTATTTGTGGGTGTCA
LUCAT1	OBS-3195	GGCAGTGAAATCATTCCAAA
LUCAT1	OBS-3196	TTCAGAACCCTTCTTTGCTA
LUCAT1	OBS-3197	CTCTACTGCTTAGAGTCAGT
LUCAT1	OBS-3198	GTGACTTAATCAAGAATAAA
LUCAT1	OBS-3199	TTACTGGGTAAAACAGCACT
None (control)	OBS-2899	CACTATGGAAAGGCGGCTTC
None (control)	OBS-2900	GATTTCGGTCTGTACGGCTA
None (control)	OBS-2901	TTACATGGTCCTAATCGGCT
None (control)	OBS-2902	GCTGTTACCTTCCACGCCGG
None (control)	OBS-2903	ATACGATCGGACAGCCTTGT
None (control)	OBS-2904	TGCACAATTGATGTTCCGAT
None (control)	OBS-2905	GACGCCTAGACGTATACTAG
None (control)	OBS-2906	GTGTGTGCTATTAGAAGCGG
None (control)	OBS-2907	AAGCGACCCTGACAGTGCGA
None (control)	OBS-2908	AGCAAACACGTCGAGCAAAT

Supplementary Table 5: Sequencing and RT-qPCR primers used in the present study.

RT-qPCR target (purpose)	DNA oligo internal ID	DNA oligo sequence
hU6 (Sanger sequencing)	OBS-0755	Fwd: GAGGGCCTATTTCCCATGATTC
hU6 (RT-qPCR)	OBS-0712	Fwd: GCTTCGGCAGCACATATACTAAAAT
	OBS-0713	Rev: ATATGGAACGCTTCACGAATTTG
hRPS18 (RT-qPCR)	OBS-107	Fwd: GCGGCGGAAAATAGCCTTTG
	OBS-108	Rev: GATCACACGTTCCACCTCATC
ROCKI (RT-qPCR)	OBS-1286a	Fwd: TGGCTGCTCTAATCCTTGACC
	OBS-1287a	Rev: AGCATGAGTTACTGTGAAACCATC
LINC00158 (RT-qPCR)	OBS-1448	Fwd: TGGCTAAAGAGACTCTGGCT
	OBS-1449	Rev: GGAGTTCTTCTGCCACCTTG
LINC01215 (RT-qPCR)	OBS-0889	Fwd: ATTCTCCTGCCTCACAAGTGC
	OBS-0890	Rev: TCTGCTCTATGTCTGCACTGG
AC010980.1 (RT-qPCR)	OBS-2594	Fwd: AGATGAATTGCCAGTATATGTTGC
	OBS-2595	Rev: AGCATACCTTCAGTAGATTCTGTG
AC022816.1 (RT-qPCR)	OBS-2879	Fwd: TCAAGAGATTCTCCTGCCTCAGAC
	OBS-2880	Rev: TCAGACTTAGGTTCTTGTCTGTCC
MalL1 (RT-qPCR)	OBS-865	Fwd: AGCTCTGAGGAGTGAATCCAC
	OBS-866	Rev: ACATGGCTTTCATGCTAAATCTGTG
hCXCL8 (IL-8) (RT-qPCR)	OBS-0017	Fwd: ACTGAGAGTGATTGAGAGTGGAC
	OBS-0018	Rev: AACCCTCTGCACCCAGTTTTC
hIFNB1 (RT-qPCR)	OBS-2204	Fwd: AACATGACCAACAAGTGTCTCC
	OBS-2205	Rev: TGTCCTTGAGGCAGTATTCAAG
hCXCL10 (RT-qPCR)	OBS-400	Fwd: CTGCCATTCTGATTTGCTGCC
	OBS-401	Rev: GATGCAGGTACAGCGTACAG
hGATA2 Exon 1-2 (RT-qPCR)	OBS-3230	Fwd: TGCTCCCAGCTCTACTCCAG
	OBS-3231	Rev: TGCTGCGCATTCAGCACG
hGATA2 Exon 2-3 (RT-qPCR)	OBS-3232	Fwd: TCTTCAATCACCTCGACTCGC
	OBS-3233	Rev: AGTGGCGTCTTGGAGAAGG
hGATA2 Exon 3-4 (RT-qPCR)	OBS-3234	Fwd: ACGACTACAGCAGCGGACTC
	OBS-3235	Rev: ACAGGCATTGCACAGGTAGTG
hGATA2 Exon 4-5 (RT-qPCR)	OBS-3236	Fwd: AATGGGCAGAACCGACCAC
	OBS-3238	Rev: TTGTAGTAGAGGCCACAGGC
hGATA2 Exon 5-6 (RT-qPCR)	OBS-3237	Fwd: TGTCAGACGACAACCACCAC
	OBS-3239	Rev: ACATCTTCCGGTTCCGAGTC
hnRNP L (RT-qPCR)	OBS-3576	Fwd: TTCTGCTTATATGGCAATGTGG
	OBS-3577	Rev: GACTGACCAGGCATGATGG
hnRNP A1 (RT-qPCR)	OBS-3590	Fwd: TCAGAGTCTCCTAAAGAGCCC
	OBS-3591	Rev: ACCTTGTGTGGCCTTGCAT
hnRNP A3 (RT-qPCR)	OBS-4278	Fwd: TGATGGGCGTGTAGTGGAAC

	OBS-4279	Rev: AGCAGACTGCATCTCTTGTTTAG
hnRNP A2B1 (RT-qPCR)	OBS-3586	Fwd: AGCTTTGAAACCACAGAAGAA
	OBS-3587	Rev: TTGATCTTTTGCTTGCAGGA
hnRNP D (RT-qPCR)	OBS-4276	Fwd: GCGTGGGTTCTGCTTTATTACC
	OBS-4277	Rev: TTGCTGATATTGTTCCTTCGACA
ROCKI (ChIRP-qPCR)	OBS-4481	Fwd: TTGGACCTTCTCATCCCAGC
	OBS-4482	Rev: GCCACAGCTGTAGTTGTTTGG
MARCKS promoter site 1 (ChIRP-qPCR)	OBS-4483	Fwd: CGACTGCTTCTCACCTTCA
	OBS-4484	Rev: TACCTCCATCTGTCACTGC
MARCKS promoter site 2 (ChIRP-qPCR)	OBS-4485	Fwd: GCAGTGACAGATGGAGGTA
	OBS-4486	Rev: GCAGCAACGATGACATTCT

Supplementary Table 6: External RNA-seq data analyzed in the present study.

Publication (PMID)	Downloaded .sra file (GSE #)	Cell type	Condition
Managò et al. (31511522)	SRR9956039 (GSE135753)	Blood-derived macrophage	control
Managò et al. (31511522)	SRR9956043 (GSE135753)	Blood-derived macrophage	control
Managò et al. (31511522)	SRR9956047 (GSE135753)	Blood-derived macrophage	control
Managò et al. (31511522)	SRR9956036 (GSE135753)	Blood-derived macrophage	6 h LPS
Managò et al. (31511522)	SRR9956040 (GSE135753)	Blood-derived macrophage	6 h LPS
Managò et al. (31511522)	SRR9956044 (GSE135753)	Blood-derived macrophage	6 h LPS
Lewis et al. (24967665)	SRR1282195 (GSE57494)	Blood-derived macrophage	control
Lewis et al. (24967665)	SRR1282196 (GSE57494)	Blood-derived macrophage	control
Lewis et al. (24967665)	SRR1282197 (GSE57494)	Blood-derived macrophage	control
Lewis et al. (24967665)	SRR1282180 (GSE57494)	Blood-derived macrophage	6 h LPS + IFNγ
Lewis et al. (24967665)	SRR1282181 (GSE57494)	Blood-derived macrophage	6 h LPS + IFNγ
Lewis et al. (24967665)	SRR1282182 (GSE57494)	Blood-derived macrophage	6 h LPS + IFNγ
Lissner et al. (26147648)	SRR1539216 (GSE60216)	Blood-derived monocyte	control
Lissner et al. (26147648)	SRR1539219 (GSE60216)	Blood-derived monocyte	control
Lissner et al. (26147648)	SRR1539222 (GSE60216)	Blood-derived monocyte	control
Lissner et al. (26147648)	SRR1539218 (GSE60216)	Blood-derived monocyte	6 h LPS
Lissner et al. (26147648)	SRR1539221 (GSE60216)	Blood-derived monocyte	6 h LPS
Lissner et al. (26147648)	SRR1539224 (GSE60216)	Blood-derived monocyte	6 h LPS

Supplementary Table 7: Antibodies used in the present study.

**Antibody description**	**Dilution**	**Source**	**Identifier**
Anti-hnRNP L antibody, mouse monoclonal (clone 4D11)	1:10000	Sigma-Aldrich	Cat# R4903
Anti-FLAG M2 antibody, mouse monoclonal	- (control IgG for CoIP)	Sigma-Aldrich	Cat# F1804
Anti-mouse-HRP, Goat IgG	1:1000	Santa Cruz	sc-2005

Supplementary Note 1: GRADR step-by-step protocol

This protocol outlines the GRADR method in four major steps: **I.** RNA-protein co-sedimentome analysis via GRAD-seq. **II.** RNA-binding protein identification via OOPS-MS. **III.** Subcellular fractionation to assign RNAs and proteins to the cytoplasm and nucleus. **IV.** GRADR data integration to predict RNA-protein interactions. The protocol is optimized for human cells but can be adapted for other species with minor modifications, such as species-specific adjustments to the RNA-seq data mapping and RT-qPCR validation strategies.

I. GRAD-seq (RNA-protein co-sedimentome analysis)

This section details the determination of global sedimentation profiles for cellular RNAs and proteins using a 10-60% glycerol gradient. Although automation is possible, this protocol is designed to be accessible to labs without specialized equipment. The protocol is optimized for the Servall Evolution Ultracentrifuge (Hitachi) using 40 ml gradient tubes. We recommend performing initial test runs (using A260 absorbance, RT-qPCR and silver staining analysis as outlined) and adjusting centrifugation time and speed if using different equipment.

GRAD-seq part A: Gradient preparation:

1. In a Styrofoam box prepare a dry-ice/ethanol slurry, dense enough to support the ultracentrifugation tubes in an upright position. Allow the ethanol to cool for ~15 min.
2. Prepare aqueous gradient buffer (10 mM Tris [pH 8], 150 mM NaCl, 10 mM KCl, 1.5 mM $MgCl_2$, 0.5 % Triton, 0.5 mM EDTA, 1 mM DTT).
3. Prepare two base solutions in gradient buffer: one with 60% glycerol and one with 0% glycerol (i.e. gradient buffer only).
4. Create intermediate glycerol solutions (10, 15, 20, 25, 30, 35, 40, 45, 50, 55%) by mixing the two base solutions according to the table below (example for 5 ml):

Final concentration	60 % Glycerol (ml)	0 % (gradient buffer) (ml)
10%	0.83	4.17
15%	1.25	3.75
20%	1.67	3.33
25%	2.08	2.92
30%	2.50	2.50
35%	2.92	2.08
40%	3.33	1.67
45%	3.75	1.25
50%	4.17	0.83
55%	4.58	0.42

5. Place a 40 ml ultracentrifugation screw cap tube into the ethanol/dry-ice slurry and add 3.5 ml of the 60% glycerol solution. Allow the solution to freeze completely.
6. Sequentially add the remaining glycerol solutions in 3.5 ml increments, starting from the next highest percentage and ending with the 10% solution. Allow each layer to freeze before adding the next.

7. Close the tube and store the prepared gradient at – 80 °C.

GRAD-seq part B: Loading, ultracentrifugation and fractionation:

1. Thaw the gradient upright at 4 °C overnight, facilitating the formation of a continuous glycerol gradient.
2. Collect ~10^8 cells (protocol optimized for blood-derived human macrophages) into a 15-50 ml Falcon tube.
3. Wash cells once with 10 ml PBS (centrifuge at 300 g for 5 min).
4. Resuspend cell pellet in 400 µl of lysis buffer (same composition as the gradient buffer).
5. Transfer the lysate to a 1.5 ml tube.
6. Incubate on ice for 10 min.
7. Pass the lysate through a 26-gauge needle five times.
8. Gently disrupt nuclei using a dounce homogenizer (8-10 strokes).
9. Transfer the homogenized lysate to a fresh 1.5 ml tube.
10. Add 2 µl of RNase inhibitor (e.g., Promega RNasin, # N2511).
11. Briefly centrifuge the lysate in a benchtop centrifuge (30 seconds at 8000 g) to pellet cellular debris.
12. Carefully layer the cleared lysate onto the top of the pre-formed glycerol gradient using a pipette.
13. Balance the tubes and load them into the ultracentrifuge. For the Servall Evolution Ultracentrifuge (Hitachi) with Servall S-34 rotor, centrifuge at 50,200 rcf, with acceleration set to level 1 and brakes turned off, for 20 h at 4 °C.

⇨ **Note:** Always ensure that the lowest acceleration level is selected and breaks are turned off, to prevent from disturbing the gradient integrity.

14. After ultracentrifugation, carefully collect 900 µl fractions from the top of the gradient using a pipette with a 1 ml tip. Maintain contact with the liquid surface through surface tension. Don't punch through the liquid surface, to ensure precision. Transfer each 900 µl fraction into a separate 2 ml tube and keep on ice.
15. Remove 100 µl from each fraction for SDS-PAGE / Western blot analysis. Store these aliquots on ice or at -80 °C for long term storage.
16. After collecting all gradient fractions, resuspend the pellet at the bottom of the tube in 900 µl of gradient buffer by pipetting repeatedly, and transfer to a new 2 ml tube. This constitutes the final fraction.

GRAD-seq part C: Protein / RNA recovery

Note: all subsequent centrifugation steps are performed using a benchtop centrifuge.

1. Add 800 µl of cold phenol-chloroform-isoamyl alcohol (PCI, "for RNA") to each fraction.
2. Vortex vigorously.
3. Centrifuge at max. speed for 30 min at 15 °C.
4. With each tube, proceed with the following two protocols using the separated phases (aqueous phase: "RNA recovery" protocol; Phenol phase: "Protein recovery" protocol).

RNA recovery:

5. Transfer 700 µl of the aqueous phase to a new 1.5 ml tube.
6. Add 700 µl PCI ("for RNA") and vortex.
7. Centrifuge at max. speed for 30 min at 15 °C.
8. Transfer 600 µl of the new aqueous phase to a fresh 2 ml tube.
9. Precipitate RNA by adding 1 µl GlycoBlue and 1.4 ml of ice-cold ethanol with 3M sodium acetate (30:1 ratio).
10. Invert the tube several times and incubate at -20 °C for at least 2 h or overnight.
11. Pellet RNA by centrifugation at max. speed for 20 min at 4 °C.
12. Carefully aspirate the supernatant and wash the pellet with 500 µl of 70% ice-cold ethanol (invert the tube 3-5 times).

13. Centrifuge at max. speed for 5 min at 4 °C, then remove the supernatant completely and air-dry the pellet.
14. Add 43.5 µl of water and incubate the tube at 65 °C for 5 min. Vortex and place the tube on ice.
15. Add 5 µl DNase I buffer (Ambion), 0.5 µl RNase inhibitor (e.g., Promega RNasin, # N2511) and 1 µl DNase I (Ambion, # EN0521).
16. Mix gently by pipetting and incubate at 37 °C for 30 min.
17. Inactivate by heating at 75 °C for 5 min.
18. Spin down briefly, then add 50 µl H_2O.
19. Transfer ~100 µl to a fresh 1.5 ml tube.
20. Add 100 µl PCI ("for RNA"), vortex and centrifuge at max. speed for 15 min at 15 °C.
21. Transfer upper phase (~80 µl) to a fresh 1.5 ml tube.
22. Precipitate RNA by adding 1 µl GlycoBlue and 300 µl of ice-cold ethanol with 3M sodium acetate (30:1 ratio).
23. Invert the tube several times and incubate at -20 °C for at least 2 h or overnight.
24. Pellet RNA by centrifugation at max. speed for 20 min at 4 °C.
25. Carefully aspirate the supernatant and wash the pellet with 500 µl of 70% ice-cold ethanol (invert the tube 3-5 times).
26. Centrifuge at max. speed for 5 min at 4 °C, then remove the supernatant completely and air-dry the pellet.
27. Add e.g. 30 µl of water and incubate the tube at 65 °C for 5 min. Vortex and place the tube on ice. Determine RNA-concentration (e.g., Nanodrop analysis).

Protein recovery:

28. For protein precipitation, first add 800 µl of H_2O to the phenol phase and vortex (this step is critical for removing excess glycerol, which can inhibit protein precipitation).
29. Centrifuge at 17,000 g for 30 min at 15 °C.
30. Carefully remove and discard the aqueous phase.
31. Add 1.2 ml of ice-cold acetone to the remaining phenol phase, mix vigorously by pipetting.
32. Incubate over night at -20 °C.
33. Centrifuge at max. speed for 30 min at 4 °C.
34. *(Optional)*: Wash pellet with 500 µl ice-cold acetone for additional purity.
35. Air-dry the pellet and resuspend in 0.5 ml mass-spectrometry-grade 8 M urea, 0.1 M ammonium bicarbonate.

⇨ **Note**: If the pellet does not fully resuspend upon incubation at room-temperature for 5 min and vortexing, perform two freeze-thaw cycles with intermittent vortexing. Should residual pellet material still be visible, proceed using the supernatant, as the majority of the protein will have already dissociated.

36. Determine protein concentration (e.g. BCA procedure).

GRAD-seq part D: Sample analysis:

We recommend assessing gradient quality by analyzing the gradient samples collected above using A260 absorbance measurements, RT-qPCR, and SDS-PAGE coupled to silver staining. The following guidelines are provided for gradients fractionated into 44 consecutive fractions. Results of the analysis outlined below from a similar GRAD-seq setup are available in Aznaourova et al., PNAS 2020, Fig. S1D (PMID: 32241891).

1. Pooling fractions to reduce sample numbers in downstream analysis

   Continuously pool fractions from the SDS-PAGE, RNA, and protein sample series stored, by combining every two successive samples (e.g. fractions 1+2, 3+4, 5+6…) to yield 22 pooled samples for each sample type. This facilitates the down-stream analysis described below.

2. Protein analysis (quality control step)

SDS-PAGE and Silver Staining:

- Each pooled sample for SDS-PAGE should be combined with a loading buffer, such as Laemmli, and boiled before loading, according to standard lab protocols.
- Load an aliquot equivalent to 15 µl of each pooled sample per well of a 10% SDS-PAGE gel. After finishing the gel run, perform protein staining using standard lab protocols. We recommend Silver Staining, which has proven to be a highly sensitive and quantitative protein visualization approach in our lab.
- Expect to observe distinct protein bands across the gradient. The majority of proteins should be concentrated in the first 3–5 pooled fractions and in the final pooled fraction (22).

3. RNA analysis (quality control step)

A260 absorbance measurement:

- Use a spectrophotometer (e.g., Nanodrop) to assess RNA distribution.
- Typically, the majority of RNA will be found in the last pooled fraction, with additional peaks appearing around pooled fractions 2–4 and 13–16.

RT-qPCR:

- Use one-step RT-qPCR kits and add equal volumes of RNA from each fraction (instead of equalizing cDNA concentrations) to accommodate differences in RNA content among the different gradient fractions.
- Analyze the distribution of specific RNA species:
    - *U6 snRNA*: Expected to peak in the first five pooled fractions.
    - *MALAT1 lncRNA*: Should show a peak around pooled fraction 10.
    - *GAPDH mRNA*: Primarily peaks in the last pooled fraction.

4. RNA-seq and proteomics

If the above controls confirm the expected distribution of proteins and RNA, continue with the following:

RNA-Seq analysis:

- Spike-In addition:
    - Take 25 µl from each pooled fraction and add 60 ng of *Escherichia coli* total RNA (prepared, for example, via glass-bead lysis followed by the Qiagen RNeasy kit protocol). This Spike-In should represent approximately 5% of the RNA content of the fraction with the lowest RNA yield (typically pooled fraction 8, corresponding to original fractions 15+16).
    - Mix thoroughly after Spike-In addition.

- Library preparation and data analysis:
    - Use ~1 µg of Spike-In supplemented RNA from each fraction to generate RNA-seq libraries with total RNA protocols (e.g., Illumina TruSeq Stranded Total RNA kit; note: no ribosomal RNA depletion, polyA enrichment, or 3′-end sequencing).
    - Map the reads to a combined human and *Escherichia coli* reference genome. Normalize human RNA counts to the *E. coli* Spike-In RNA counts to account for differences in RNA content across fractions, as described in Aznaourova et al., PNAS 2020 (PMID: 32241891).

Proteomics analysis:

- Protein quantification:
  - Determine the protein concentration in each pooled fraction using a BCA assay.
  - Supplement each pooled protein sample with a fixed amount of Spike-In protein (e.g., cytochrome C, corresponding to 5% of the total protein in the fraction with the lowest concentration).
- Proteomics run and data analysis:
  - Perform tryptic digest and label-free MS/MS quantifications using instrument-specific protocols. We recommend using MaxQuant for data analysis.
  - Normalize the obtained abundance values (e.g. LFQ-values) either based on the Spike-In or on the total protein content in each fraction, as detailed in Aznaourova et al., PNAS 2020 (PMID: 32241891).

5. <u>Data analysis and integration</u>

Normalization and expression metrics:

- Import normalized RNA and protein abundance values (e.g., normalized RPKMs for RNA and LFQs for proteins) into a spreadsheet (e.g., rows = genes, columns = gradient fractions [1-22]).
- Express each value as either a fold change over the row base-mean or as a row Z-score. This will generate comparable gradient distribution metrics for RNA and protein.
- These normalized values can now be used to perform RNA-protein co-sedimentation analyses, e.g. based on Pearson correlation r- and p-values, to predict RNA–protein interactions (see part IV, GRADR data integration).

II. RNA-binding protein identification via OOPS-MS

This section describes a proteome-wide approach to assess the RNA-binding potential of proteins using the Orthogonal Organic Phase Separation (OOPS) method as described by Villanueva et al. (Nat Protoc 2020, PMID: 32651564). In this adapted protocol, we employ an RNase-based elution strategy to specifically release RNA-bound proteins. The resulting data refine GRAD-seq predictions by focusing on proteins with significant RNA affinity.

OOPS-MS part A: UV crosslinking and cell lysis:

1. Seed 3 x 10^6 cells into a 90 mm dish and differentiate/stimulate as required.
2. Remove the medium and wash the cells twice with an equivalent volume of PBS.
3. Remove any residual PBS, place the dish on ice and immediately proceed to crosslinking.
4. UV-irradiate the cells on ice at 400 mJ/cm^2.
5. Add 1 ml of TRIzol directly to the dish.
6. Scrape the dish thoroughly and transfer the crosslinked cell lysate to a 1.5 ml tube.
7. Pipette up and down five times, then vortex briefly.
8. Incubate at room temperature for 5 min.

OOPS-MS part B: RNA binding protein enrichment and controls

Phase separation:

1. Split the lysate into two tubes.
2. To each tube add 150 µl chloroform.
3. Vortex for 15 s.
4. Centrifuge for 15 min at 12,000 g, 4 °C.
5. Carefully remove the upper aqueous phase.
6. With the pipette tip, pass through the interface to collect the lower organic phase, transfer this organic phase to a tube labeled “free protein”.
7. Combine the interphases from both tubes.
8. Add 1 ml TRIzol to the combined interphase.
9. Vortex for 15 s (or until the interphase has completely homogenized).
10. Add 200 µl chloroform and vortex for 15 s.
11. Centrifuge for 15 min at 12,000 g, 4 °C
12. Discard both the upper aqueous phase and the lower organic phase, leaving only the interphase.
13. Repeat step 8-12 to further purify the interphase.

Recovery of RNA-binding proteins:

1. To the purified interphase, add 900 µl methanol.
2. Invert the tube three times and vortex for 15 s.
3. Centrifuge for 10 min at 14,000 g, 4°C.
4. Completely remove and discard the supernatant.
5. (Optional): For increased purity, add 1 ml of methanol, repeat centrifugation and discard the supernatant.
6. Air-dry the pellet for ~2 min.
7. To the pellet add 200 µl of 100 mM Triethylammonium bicarbonate (TEAB) buffer (1 M, pH 8.5; Sigma-Aldrich, #T708) and 10 µl of 20% SDS solution. Vortex to resuspend.
8. Divide the sample equally into two tubes labeled “RNase+” and “RNase-“ (100 µl each).
9. Only “RNase+” sample: Sonicate in a cool water bath for 15 min using high setting with 30-second on/off cycles.
10. Only “RNase+” sample: Incubate at 95 °C for 20 min.
11. Only “RNase+” sample: Cool on ice for 2 min.
12. Only “RNase+” sample: Add 1 µl of RNase A/T1 mix (Thermo Fisher Scientific, #EN0551) and incubate at 37 °C for 4 h.
13. In parallel, incubate the “RNase-“ sample at 37 °C for 8 h without adding RNase.
14. Only “RNase+” sample: After the initial incubation, add an additional 2 µl of RNase A/T1 mix and incubate at 37 °C for another 4 h.
15. Add 1 ml of TRIzol to both the “RNase+” and “RNase-“ samples.
16. Vortex for 15 s.
17. Add 200 µl of chloroform and vortex for 15 s.
18. Centrifuge for 15 min at 12,000 g, 4 °C.
19. Discard the upper aqueous phase and the interface.
20. Transfer 150 µl of the organic phase to a new protein low-binding tube.
21. Add 1350 µl of methanol, vortex for 15 s and centrifuge for 10 min at 12,000 g, 4 °C.
22. Remove the supernatant (the pellet may be very small).
23. Repeat Steps 20-22 until all the collected organic phase is processed; use the same tube for all rounds.
24. Air-dry the final pellet.
25. Add 50 µl mass-spectrometry-grade 8 M urea, 0.1 M ammonium bicarbonate.
26. Vortex briefly.
27. Store sample at -20 °C over-night or until proteomics measurement.
28. Thaw and vortex until the pellet is fully dissolved.

⇨ **Note**: If the pellet does not fully resuspend upon incubation at room-temperature for 5 min and vortexing, perform two freeze-thaw cycles with intermittent vortexing. Should residual pellet material still be visible, proceed using the supernatant, as the majority of the protein will have already dissociated.

29. Transfer the RNase+ and RNase- samples, along with the "free protein" samples (see below) to mass spectrometry.

Recovery of free protein:

1. From the tube labeled "free protein", transfer 150 µl to a new protein low-binding tube.
2. Add 1350 µl of methanol, vortex for 15 s and centrifuge for 10 min at 12,000 g, 4 °C.
3. Discard the supernatant (the pellet may be very small).
4. Repeat Steps 1-3 until all the organic phase is processed; use the same tube for all rounds.
5. Air-dry the final pellet.
6. Add 50 µl mass-spectrometry-grade 8 M urea, 0.1 M ammonium bicarbonate.
7. Vortex briefly.
8. Store sample at -20 °C over-night or until proteomics measurement.
9. Thaw and vortex until the pellet is fully dissolved.

⇨ **Note**: If the pellet does not fully resuspend upon incubation at room-temperature for 5 min and vortexing, perform two freeze-thaw cycles with intermittent vortexing. Should residual pellet material still be visible, proceed using the supernatant, as the majority of the protein will have already dissociated.

10. Hand over this sample along with the "RNase+" and "RNase-" samples to mass-spectrometry.

OOPS-MS part C: Sample analysis:

Proteomics run:

1. Perform tryptic digestion and label-free MS/MS quantification according to instrument-specific protocols. We recommend using MaxQuant for data analysis.
2. Continue with the label-free quantification values (LFQ-values) obtained.

Data analysis:

1. Import the protein LFQ values into a spreadsheet (e.g., rows = genes, columns = conditions: free protein, RNase+, RNase-).
2. Express each value either as a fold change over the row base-mean or as a row Z-score to generate comparable metrics for assessing protein affinity to RNA.
3. Plot heatmaps (e.g. using row Z-scores) for selected protein groups to visualize their distribution across the free protein, RNase+, and RNase- samples.
4. Validate the success of the method by confirming that known RNA-binding proteins (e.g. hnRNPs) are enriched in the RNase+ fraction, while non-RNA-binding proteins (e.g. Golgi proteins) are primarily found in the free protein fraction.
5. If these validations are successful, proceed with the following calculations:
    - Since many strong RNA-binding proteins may have an LFQ value of 0 in the RNAse- condition, add a value of 0.1 to all LFQ values in the dataset. Calculate the ratio of averaged LFQ(+0.1) values from the RNAse+ and RNase- conditions; this fold-change indicates the shift of a protein into the organic phase upon RNase treatment.
    - Compute a p-value for each protein comparing the RNAse+ and RNase- conditions. We recommend using Perseus software to perform a t-test after log2-transformation of the original LFQ values.
    - Consider proteins with a fold-change ≥ 2, and a p-value ≤ 0.05 as potential RNA-binding proteins.

III. Cytoplasm-nucleus fractionation

This section details the procedures to separate cytoplasmic and nuclear compartments for subsequent RNA-seq and proteomics analysis. This fractionation adds additional resolution to GRADR predictions (for example, a protein is considered an interactor of a cytoplasmic RNA only if it is sufficiently detected in the cytoplasm).

Cytoplasm-nucleus fractionation part A: RNA samples:

1. Collect 5 x 10^6 cells (protocol optimized for blood-derived human macrophages) in a 15 ml Falcon tube.
2. Wash cells once with 10 ml PBS (centrifuge at 300 g for 5 min).
3. Resuspend cells in 200 µl lysis buffer (10 mM Tris pH 8; 140 mM NaCl; 1.5 mM $MgCl_2$; 0.5% Igepal, 2 mM vanadyl ribonucleoside complex).
4. Transfer lysate to a 1.5 ml tube and incubate on ice for 5 min. At 2.5 min gently pipette up and down a few times.
5. Centrifuge at 1000 g for 3 min at 4 °C to pellet the nuclei.
6. Transfer the supernatant (cytosolic fraction) to a new 1.5 ml tube. Keep the pellet (nuclear fraction) on ice.
7. Centrifuge the cytosolic supernatant at 20,000 g for 10 min at 4 °C and transfer the supernatant to a new tube (this is your clean cytosolic fraction).
8. Carefully wash the nuclear pellet twice with 160 µl lysis buffer, followed by one wash with lysis buffer containing 0.05% deoxycolic acid. For each wash, centrifuge at at 1000 g for 3 min at 4 °C.
   ⇨ **Note**: Nuclei become fragile during treatment with deoxycolic acid.
9. Resuspend the purified nuclei in 100 µl lysis buffer.
10. Add 0.5 ml TRIzol to both the purified nuclear and cytoplasmic fractions and perform RNA extraction according to standard lab protocols.
37. Resuspend the purified RNA in 43.5 µl of water (with incubation at 65 °C for 5 min). Vortex, and then place the tubes on ice.
11. Perform DNase I digestion as described in *GRAD-seq part C* above.
12. Resuspend the DNase I treated RNA in 50 µl of water (with 5 min incubation at 65 °C, followed by vortexing). Place on ice and determine RNA-concentration (e.g., by Nanodrop).
   ⇨ **Note**: Do not adjust RNA concentrations between nuclear and cytoplasmic fractions prior to Spike-In RNA addition (see below), as this may obscure the localization information in subsequent RNA-seq and RT-qPCR analysis.

Cytoplasm-nucleus fractionation part B: protein samples:

1. Collect 5 x 10^6 cells (protocol optimized for blood-derived human macrophages) in a 15 ml Falcon tube.
2. Wash cells once with 10 ml PBS (centrifuge at 300 g for 5 min).
3. Resuspend cells in 400 µl buffer A (10 mM Hepes pH 7.9, 10 mM KCl, 0.1 mM EDTA, 0.1 mM EGTA, 0.5 mM DDT, 1x cOmplete EDTA-free protease inhibitor [Roche]).
4. Incubate on ice for 15 min.
5. Pass the cells through a 26-gauge needle 7-8 times.
6. Transfer the lysate to a 1.5 ml tube and centrifuge at 5000 g for 2 min.
7. Transfer the supernatant (cytosolic fraction) to a new 1.5 ml tube. Keep the pellet (this is the nuclear fraction) on ice.

8. Centrifuge the cytosolic fraction at 20,000 g for 20 min, then transfer the supernatant to a new 1.5 ml tube (this is your clean cytosolic fraction). Keep on ice.
9. Wash the nuclear pellet obtained in step 7 twice with 200 µl buffer A (centrifuge at 5000 g for 2 min each).
10. Resuspend the washed nuclear pellet in 100 µl buffer B (20 mM Hepes pH 7.9, 400 mM NaCl, 1 mM EDTA, 1 mM EGTA, 0.5 mM DDT, 1x cOmplete EDTA-free protease inhibitor [Roche]).
11. Incubate for 1 h at 4 °C with gentle shaking.
12. Centrifuge at 20,000 g for 20 min at 4 °C and transfer supernatant to a new 1.5 ml tube (this is your clean nuclear fraction).
13. Precipitate proteins from both the clean cytosolic and nuclear fractions by adding 1 ml of ice-cold acetone.
14. Mix by inverting the tubes several times.
15. Incubate over night at -20 °C.
16. Centrifuge at max. speed for 30 min at 4 °C.
17. *(Optional)*: Wash pellet with 500 µl ice-cold acetone for additional purity.
18. Air-dry the pellet and resuspend in 50 µl of mass-spectrometry-grade 8 M urea, 0.1 M ammonium bicarbonate.
    - ⇨ **Note**: If the pellet does not fully resuspend upon incubation at room-temperature for 5 min and vortexing, perform two freeze-thaw cycles with intermittent vortexing. Should residual pellet material still be visible, proceed using the supernatant, as the majority of the protein will have already dissociated.
19. Determine protein concentration (e.g. BCA procedure).
    - ⇨ **Note**: Do not adjust protein concentrations between nuclear and cytoplasmic fractions prior to Spike-In addition (see below), as this may obscure the localization information in subsequent analysis (i.e. Western blot).

Cytoplasm-nucleus fractionation part C: Sample analysis:

1. RNA analysis (quality control step)

   RT-qPCR:
   - Use one-step RT-qPCR kits and add equal volumes of the cytoplasmic and nuclear fractions (do not equalize RNA concentrations) to accommodate differences in total RNA content.
   - Assess the distribution of the following RNA species:
     - *U6 snRNA*: Expected to display ~ 60 % nuclear localization.
     - *MALAT1 lncRNA*: Expected to display ≥ 90 % nuclear localization.
     - *B-Actin mRNA*: Expected to display ≥ 80 % cytoplasmic localization.

2. Protein analysis (quality control step)

   SDS-PAGE and Western blot:
   - Each sample for SDS-PAGE should be combined with a loading buffer, such as Laemmli, and boiled before loading, according to standard lab protocols.
   - Load an aliquot corresponding to ~5 µl of the cytoplasmic and nuclear fractions into separate wells of a 10-14% SDS-PAGE gel.
   - Following electrophoresis perform Western blot analysis using antibodies against Tubulin (for cytoplasmic validation) and Lamin (for nuclear validation).

3. RNA-seq and proteomics

   If quality control confirms the expected distribution of RNAs and proteins, proceed with downstream analysis:

RNA-Seq analysis:

- Spike-In addition:
  - Take 30 µl from each fraction (cytoplasm and nucleus) and add equal amounts of *Escherichia coli* total RNA (prepared, for example, via glass-bead lysis followed by the Qiagen RNeasy kit protocol). The amount of Spike-In RNA should equal 5% of the RNA present in the 30 µl nuclear fraction sample.
  - Mix thoroughly after Spike-In addition.

- Library preparation and data processing:
  - Use ~1 µg of Spike-In-supplemented RNA from each fraction to generate RNA-seq libraries (e.g., Illumina TruSeq Stranded mRNA kit with polyA-RNA enrichment or ribosomal RNA depletion; avoid 3'-end sequencing protocols to ensure full-length transcript coverage).
  - Map the reads to a combined human and *Escherichia coli* reference genome. Normalize human RNA counts to the *E. coli* Spike-In RNA counts to account for differences in RNA content.

Proteomics analysis

- Protein quantification:
  - Determine the protein concentration in each fraction using a BCA assay.
  - Supplement each protein sample with a fixed amount of Spike-In protein (e.g., cytochrome C, corresponding to 5% of the total protein in the nuclear fraction).
- Proteomics run and data processing:
  - Perform tryptic digestion and label-free MS/MS quantification following instrument-specific protocols. We recommend using MaxQuant for data analysis.
  - Normalize the obtained abundance values (e.g. LFQ-values) based on the Spike-In.

4. <u>Data analysis and integration</u>

Annotation of cytoplasmic and nuclear RNAs and proteins:

- Import Spike-In normalized RNA and protein abundance values (e.g. normalized RPKMs and LFQs) into a spreadsheet (e.g., rows = genes, columns = cytoplasmic and nuclear fractions).
- Calculate the percentage localization (cytoplasmic versus nuclear) for each RNA and protein based on the normalized abundance values.
- Generate a list of proteins that are consistently detected in the cytoplasmic fraction (i.e., proteins with LFQ values present in all cytoplasmic replicates and not showing ≥ 60% nuclear localization in all replicates.
- Conversely, generate a list of proteins for the nuclear fraction (i.e. proteins with LFQ values in all nuclear replicates and not showing ≥ 60% cytoplasmic localization). We recommend removing mitochondrial proteins from this dataset due to the implausible interactions between nuclear and mitochondrial factors.
- Apply a similar strategy for RNAs, using a cutoff (e.g., RPKM ≥ 0.5 before Spike-In normalization) to filter all RNAs detected in the respective fractions and the same ≥ 60% nuclear or cytoplasmic filters applied to the protein datasets.
- These curated lists can now be used to focus the RNA-protein co-sedimentation analysis described above on interactors present in the same subcellular compartment (cytoplasm or nucleus), as further detailed below.

IV. GRADR data integration (prediction of RNA-protein-interactions)

This section outlines the process for consolidating GRAD-seq, OOPS-MS, and cytoplasm/nucleus fractionation data to predict and rank proteins that likely interact with a specific RNA (e.g., a lncRNA of interest). For this purpose, the data obtained after following the data analysis instructions at the end of the GRAD-seq, OOPS-MS, and cytoplasm/nucleus fractionation protocols are combined and processed as described below.

Data integration part A: Consolidation of GRAD-seq, OOPS-MS and fractionation data:

In this part, the GRAD-seq, OOPS-MS and cytoplasm/nucleus fractionation data are consolidated into a single spreadsheet (see also **GRADR Protocol Figure 1**):

1. Create a column listing the gene names for all proteins, and include the gene name for the lncRNA of interest at the end.
2. In subsequent columns, enter the row Z-scores for the GRAD-seq profiles of each protein as well as the lncRNA.
3. Add columns for the OOPS-MS fold-change values and corresponding p-values for each protein.
4. Append columns that annotate each protein (and the lncRNA) as nuclear and/or cytoplasmic. Use a binary notation (1 if the factor is present in the compartment, 0 if absent).

	GRAD-seq profile	OOPS-MS		C/N fractionation		Pearson correlation
Identifier	row Z-sores	fold-change	p-value	nuclear	cytoplasmic	r value
Protein A		1,96E+01	1,18E-02	1	1	
Protein B		1,37E+06	3,40E-05	1	1	
Protein C		8,44E+05	1,17E-01	0	1	
Protein D		2,08E+01	2,30E-03	1	1	
Protein E		1,29E+07	3,20E-06	1	1	
Protein F		1,41E+01	2,25E-01	1	0	
Protein G		3,20E+07	1,11E-05	1	1	
Protein H		1,98E+01	7,79E-04	1	1	
Protein I		1,03E+07	2,21E-06	1	0	
Protein J		2,56E+06	3,30E-08	0	0	
Protein K		7,89E+06	4,07E-09	1	1	
Protein L		3,86E+01	6,32E-05	1	1	
LncRNA X				1	0	

GRADR Protocol Figure 1. Schematic layout of the integrated spreadsheet, displaying the various data types collected for GRADR predictions.

Data integration part B: Pearson correlation analysis:

In this step, the data table is sorted based on the similarity of the GRAD-seq profile of each protein to that of the lncRNA of interest, thereby facilitating the identification of potential interactors.

1. In a new column, calculate the Pearson correlation coefficient (r) for every protein by comparing its GRAD-seq profile to that of the lncRNA of interest (**GRADR Protocol Figure 2**). This produces an updated table containing a Pearson r value for every protein, but not yet ranked according to these values (**GRADR Protocol Figure 3**).

2. Sort the table in descending order based on the Pearson r values to prioritize proteins with profiles that most closely resemble that of the lncRNA of interest (**GRADR Protocol Figure 4**).

	GRAD-seq profile	OOPS-MS		C/N fractionation		Pearson correlation
Identifier	row Z-sores	fold-change	p-value	nuclear	cytoplasmic	r value
Protein A		1,96E+01	1,18E-02	1	1	0,017
Protein B		1,37E+06	3,40E-05	1	1	
Protein C		8,44E+05	1,17E-01	0	1	
Protein D		2,08E+01	2,30E-03	1	1	
Protein E		1,29E+07	3,20E-06	1	1	
Protein F		1,41E+01	2,25E-01	1	0	
Protein G		3,20E+07	1,11E-05	1	1	
Protein H		1,98E+01	7,79E-04	1	1	
Protein I		1,03E+07	2,21E-06	1	0	
Protein J		2,56E+06	3,30E-08	0	0	
Protein K		7,89E+06	4,07E-09	1	1	
Protein L		3,86E+01	6,32E-05	1	1	
LncRNA X				1	0	

GRADR Protocol Figure 2. Illustration of the relevant comparisons for Pearson correlation analysis. Here, the GRAD-seq profile of Protein A is compared to that of lncRNA X (indicated by an arrow) to compute a Pearson r value. This comparison is performed for all proteins relative to lncRNA X.

	GRAD-seq profile	OOPS-MS		C/N fractionation		Pearson correlation
Identifier	row Z-sores	fold-change	p-value	nuclear	cytoplasmic	r value
Protein A		1,96E+01	1,18E-02	1	1	0,017
Protein B		1,37E+06	3,40E-05	1	1	0,005
Protein C		8,44E+05	1,17E-01	0	1	0,554
Protein D		2,08E+01	2,30E-03	1	1	0,316
Protein E		1,29E+07	3,20E-06	1	1	0,026
Protein F		1,41E+01	2,25E-01	1	0	0,576
Protein G		3,20E+07	1,11E-05	1	1	0,755
Protein H		1,98E+01	7,79E-04	1	1	0,754
Protein I		1,03E+07	2,21E-06	1	0	0,757
Protein J		2,56E+06	3,30E-08	0	0	0,004
Protein K		7,89E+06	4,07E-09	1	1	-0,003
Protein L		3,86E+01	6,32E-05	1	1	0,883
LncRNA X				1	0	

GRADR Protocol Figure 3. Representation of the GRADR data table with all Pearson r values calculated, prior to sorting in descending order.

	GRAD-seq profile	OOPS-MS		C/N fractionation		Pearson correlation
Identifier	row Z-sores	fold-chang	p-value	nuclear	cytoplasmi	r value
Protein L		3,86E+01	6,32E-05	1	1	0,883
Protein I		1,03E+07	2,21E-06	1	0	0,757
Protein G		3,20E+07	1,11E-05	1	1	0,755
Protein H		1,98E+01	7,79E-04	1	1	0,754
Protein F		1,41E+01	2,25E-01	1	0	0,576
Protein C		8,44E+05	1,17E-01	0	1	0,554
Protein D		2,08E+01	2,30E-03	1	1	0,316
Protein E		1,29E+07	3,20E-06	1	1	0,026
Protein A		1,96E+01	1,18E-02	1	1	0,017
Protein B		1,37E+06	3,40E-05	1	1	0,005
Protein J		2,56E+06	3,30E-08	0	0	0,004
Protein K		7,89E+06	4,07E-09	1	1	-0,003
LncRNA X				1	0	

GRADR Protocol Figure 4. GRADR data table after sorting by Pearson r values in descending order.

Data integration part C: Filtering steps to highlight the most likely interactors:

In this step, multiple filters are applied to the data table, to focus the GRADR predictions on proteins that exhibit significant RNA-binding potential, localize to the same subcellular compartment (cytoplasm or nucleus), and display a GRAD-seq profile similar to that of the lncRNA of interest (see **GRADR Protocol Figure 5**).

1. In the OOPS-MS data columns, retain only proteins with an OOPS-MS fold-change value ≥ 2, with a p-value ≤ 0.05.
2. Filter the table to display only those proteins that localize to the same subcellular compartment as the lncRNA. In the example provided in GRADR Protocol Figure 5, the lncRNA is primarily nuclear (> 50 %) and therefore the C/N fractionation columns are filtered to retain only proteins annotated as nuclear (value of 1 in the "nuclear" column).
3. To focus the predictions on proteins with similar GRAD-seq profiles as the lncRNA of interest, further filter the table by removing proteins with Pearson r values < 0.5.

The remaining proteins are considered potential interactors of the lncRNA of interest. We recommend performing pathway analysis on the top 20 predicted interactors to identify relevant cellular processes and machineries the lncRNA may interact with. These data can refine interactor predictions made by other methods (e.g., targeted ChIRP-MS or computational predictions based on ENCODE CLIP-seq data) and serve as a foundation for further functional studies of the lncRNA.

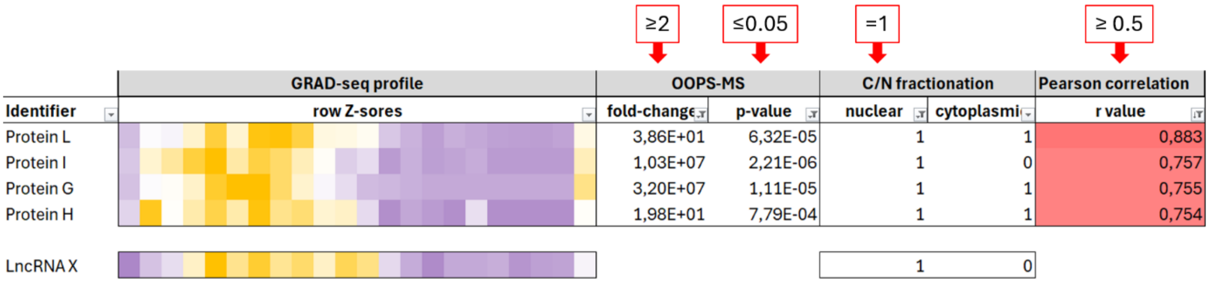

	GRAD-seq profile	OOPS-MS		C/N fractionation		Pearson correlation
		≥2	≤0.05	=1		≥ 0.5
Identifier	row Z-sores	fold-change	p-value	nuclear	cytoplasmi	r value
Protein L		3,86E+01	6,32E-05	1	1	0,883
Protein I		1,03E+07	2,21E-06	1	0	0,757
Protein G		3,20E+07	1,11E-05	1	1	0,755
Protein H		1,98E+01	7,79E-04	1	1	0,754
LncRNA X				1	0	

GRADR Protocol Figure 5. Visualization of the filtered GRADR data table, showing proteins that meet the OOPS-MS, cytoplasm/nucleus fractionation (C/N) and Pearson correlation criteria indicated (red boxes and arrows).
