# Supplementary material for: A searchable atlas of pathogen-sensitive lncRNA networks in human macrophages
Source: Nat Commun. 2025 May 21;16:4733. doi: 10.1038/s41467-025-60084-x (PMC12095776; doi:10.1038/s41467-025-60084-x)
Supplement: Supplementary file 2 — Description of Additional Supplementary Files [file 41467_2025_60084_MOESM2_ESM.pdf]

Description of Additional Supplementary Files

Supplementary Data 1: RNA-seq profiles of AECII, aMΦ, G-MΦ and M-MΦ. RPKM values and fold-changes (flagellin- or LPS-stimulation vs mock control) are provided for all genes detected in any of the four cell types under at least one treatment condition (RPKM value ≥ 0.5 in both replicates).

Supplementary Data 2: RNA-seq profile of G-MΦ, upon different immune stimuli and time-points. RPKM values and fold-changes (Pam3CSK4, IFNα and LPS-stimulation vs 0.5 h control) are provided for all genes detected in any of the control or stimulation conditions (RPKM value ≥ 0.5 in both replicates).

Supplementary Data 3: RNA-seq profile of G-MΦ, upon treatment with pathway inhibitors. RPKM values and fold-changes (LPS-stimulation alone or with inhibitor pre-incubation vs control) are provided for all genes detected in any of the control or stimulation conditions (RPKM value ≥ 0.5 in both replicates). Since not all LPS-inducible lncRNAs in the focus of this study passed the RPKM filter in this dataset, the pertaining lncRNAs were included without the RPKM ≥ 0.5 criterium.

Supplementary Data 4: RNA-seq profiles of THP1 MΦs upon lncRNA knockdown. RPKM values and fold-changes (LPS-stimulation versus mock or lncRNA CRISPRi versus control CRISPRi) are provided for all genes detected in any of the stimulation or knockdown conditions (RPKM value ≥ 0.5 in all replicates). Additionally, DeSeq2 statistics are provided for all comparisons. Since not all LPS-inducible lncRNAs in the focus of this study passed the RPKM filter in this dataset, the pertaining lncRNAs were included without the RPKM ≥ 0.5 criterium.

Supplementary Data 5: Significant RNA and protein changes in THP1 MΦ, upon lncRNA knockdown. Base-mean fold-changes of RNA and protein expression in lncRNA-silenced compared to control cells are shown (for the five lncRNAs in the focus of this study). The dataset entails all genes significantly (p ≤ 0.05) regulated at both the RNA and protein level upon silencing of at least one of the five lncRNAs.

Supplementary Data 6: G-MΦ RNA-bound proteome (OOPS-MS) dataset. iBAQ and LFQ abundance values for proteins detected in the input, elutions with RNase-treatment (RNase+) or control elutions without RNase-treatment (RNase-) from three independent experiments with lysates of LPS-treated G-MΦs are provided. Proteins marked as potential sample contaminants were removed from the dataset. Fold-changes and p-values indicating RNA-binding potential were calculated based on the LFQ abundance values (two-tailed Student's t-test; RNase+ vs RNase- LFQs).

Supplementary Data 7: G-MΦ fractionation RNA-seq and proteome dataset. "RNA-seq" tab: Spike-in adjusted RPKM values from cytoplasmic and nuclear fractions and percentage of RNA localization to both compartments. Two experimental replicates. "Proteomics" tab: normalized LFQ abundance values for cytoplasmic and nuclear fractions, as well as percentages of cytoplasmic and nuclear localization are shown. Detection of the proteins in all three experiments in the cytoplasmic and nuclear fraction, respectively, is indicated in the "Compartment" columns ("1"). Only, where proteins reached ≥ 60 % in all three replicates for the cytoplasmic or nuclear compartment, the protein was exclusively assigned to this compartment.

Supplementary Data 8: G-MΦ GRADR dataset. "Protein_dataset" tab: Grad-MS data: Grad-seq-determined distribution of proteins along a 10-60 % linear glycerol gradient (fraction 1-22), expressed as row Z-score, calculated from LFQ abundance values. OOPS-MS data: RNA-binding potential of proteins based on the OOPS-MS dataset (Supplementary Data 6) (fold-change compared to control elution and p-value). C_N_fractionation: Presence of the proteins in the nucleus and cytoplasm indicated (1 = present, 0 = not present, #N/A: not determined), determined by nucleus and cytoplasm fractionation followed by protein mass-spectrometry (Supplementary Data 7). "RNA_dataset" tab: Grad-seq-determined distribution of RNAs along a 10-60 % linear glycerol gradient (fraction 1-22), expressed as row Z-score, calculated from spike-in normalized read counts. Remaining columns: Presence of the RNAs in the nucleus and cytoplasm in %, determined by nucleus and cytoplasm fractionation followed by RNA-seq (Supplementary Data 7).

Supplementary Data 9: LUCAT1- and ROCKI-ChIRP-MS dataset. "LUCAT1 ChIRP-MS" tab: LFQ abundance values for proteins detected in the control (Ctrl) and LUCAT1 elutions from three independent ChIRP experiments (each with two technical replicates), using LPS-treated G-MΦs. Proteins marked as potential sample contaminants were removed from the dataset. Fold-changes and p-values indicating protein-binding to LUCAT1 were calculated based on the LFQ abundance values (two-tailed Student's t-test; LUCAT1 vs control LFQs). "ROCKI ChIRP-MS" tab: same as LUCAT1 tab, but showing ROCKI ChIRP-MS data obtained with LPS-treated THP1-MΦs.

Supplementary Data 10: hnRNP L CLIP-MS dataset. iBAQ abundance values for proteins detected in the control (Ctrl) and hnRNP L elutions from two independent CLIP experiments, using lysates of LPS-treated THP1-MΦs are provided. Proteins marked as potential sample contaminants were removed from the dataset. Fold-changes indicating protein-binding to hnRNP L were calculated based on the iBAQ abundance values (hnRNP L vs control iBAQs) and dataset was sorted based on the mean iBAQs from the hnRNP L CLIPs.

Supplementary Data 11: GATA2 overexpression dataset. LFQ abundance values for proteins detected in control (C) and GATA2 overexpressing THP1-MΦs, either mock-treated or challenged with LPS are provided (three experimental replicates). Proteins marked as potential sample contaminants were removed from the dataset. Fold-changes and p-values (two-tailed Student's t-test) indicating protein regulation upon GATA2 overexpression were calculated based on the LFQ abundance values (GATA2 LPS vs C LPS) and dataset was sorted based on the obtained fold-change values.

Supplementary Data 12: Grad-seq profile of major protein machinery components and lncRNAs. Row Z-score values for proteins belonging to the indicated cellular machineries and for the five lncRNAs in the focus of this study are provided, illustrating their relative abundances in 22 consecutive fractions of a linear glycerol gradient. Row Z-scores were calculated based on normalized LFQ (proteins) or RPKM (lncRNAs) values (see Aznaourova et al., PNAS 2020).
