# Supplementary material for: A searchable atlas of pathogen-sensitive lncRNA networks in human macrophages
Source: Nat Commun. 2025 May 21;16:4733. doi: 10.1038/s41467-025-60084-x (PMC12095776; doi:10.1038/s41467-025-60084-x)
Supplement: Supplementary file 15 — Reporting Summary [file 41467_2025_60084_MOESM15_ESM.pdf]

Corresponding author(s): Leon N Schulte

Last updated by author(s): Apr 28, 2025

Reporting Summary

Nature Portfolio wishes to improve the reproducibility of the work that we publish. This form provides structure for consistency and transparency in reporting. For further information on Nature Portfolio policies, see our Editorial Policies and the Editorial Policy Checklist.

Statistics

For all statistical analyses, confirm that the following items are present in the figure legend, table legend, main text, or Methods section.

n/a	Confirmed	
☐	☒	The exact sample size (n) for each experimental group/condition, given as a discrete number and unit of measurement
☐	☒	A statement on whether measurements were taken from distinct samples or whether the same sample was measured repeatedly
☐	☒	The statistical test(s) used AND whether they are one- or two-sided<br>*Only common tests should be described solely by name; describe more complex techniques in the Methods section.*
☐	☒	A description of all covariates tested
☐	☒	A description of any assumptions or corrections, such as tests of normality and adjustment for multiple comparisons
☐	☒	A full description of the statistical parameters including central tendency (e.g. means) or other basic estimates (e.g. regression coefficient) AND variation (e.g. standard deviation) or associated estimates of uncertainty (e.g. confidence intervals)
☐	☒	For null hypothesis testing, the test statistic (e.g. F, t, r) with confidence intervals, effect sizes, degrees of freedom and P value noted<br>*Give P values as exact values whenever suitable.*
☒	☐	For Bayesian analysis, information on the choice of priors and Markov chain Monte Carlo settings
☒	☐	For hierarchical and complex designs, identification of the appropriate level for tests and full reporting of outcomes
☐	☒	Estimates of effect sizes (e.g. Cohen's d, Pearson's r), indicating how they were calculated

Our web collection on statistics for biologists contains articles on many of the points above.

Software and code

Policy information about availability of computer code

Data collection	Sratoolkit v3.1.1, CLC Worbench v 10.0.1, MaxQuant v2.6, Perseus v 2.1, RBPBench v0.8.1, GraphProt v1.1.7 and RNAProt v0.5, bedtools v2.31.1, LEGENDplex Software Suite
Data analysis	Graphpad PRISM v6, MS Office Professional Plus 2024, Cluster v3.0, Java TreeView v1.2, Cytoscape v3.7.2, R Studio v4.4.2 (DeSeq2 and rgl package), Integrative Genomics Viewer v2.1.6,

For manuscripts utilizing custom algorithms or software that are central to the research but not yet described in published literature, software must be made available to editors and reviewers. We strongly encourage code deposition in a community repository (e.g. GitHub). See the Nature Portfolio guidelines for submitting code & software for further information.

Data

Policy information about availability of data

All manuscripts must include a data availability statement. This statement should provide the following information, where applicable:

- Accession codes, unique identifiers, or web links for publicly available datasets
- A description of any restrictions on data availability
- For clinical datasets or third party data, please ensure that the statement adheres to our policy

The high throughput sequencing data generated in this study have been deposited in the NCBI GEO database and are available under the accession codes GSE268546 (ChIRP-seq data; https://www.ncbi.nlm.nih.gov/geo/query/acc.cgi?&acc=GSE268546) and GSE268547 (RNA-seq data; https://www.ncbi.nlm.nih.gov/geo/query/acc.cgi?acc=GSE268547).

The mass spectrometry proteomics data generated in this study have been deposited in the ProteomeXchange Consortium PRIDE 53 repository and are available under the accession code PXD061457 (http://proteomecentral.proteomexchange.org/cgi/GetDataset?ID=PXD061457):

Key omics data obtained as part of this study are available as supplementary data files and through the SMyLR web interface (rna-lab.org/smylr). All data are included in the Supplementary Information or available from the authors, as are unique reagents used in this Article. The raw numbers for charts and graphs are available in the Source Data file whenever possible. Source Data are provided with this paper.

Research involving human participants, their data, or biological material

Policy information about studies with human participants or human data. See also policy information about sex, gender (identity/presentation), and sexual orientation and race, ethnicity and racism.

Reporting on sex and gender	This study did not explicitly perform sex-based analysis, as this was not in the focus of the present manuscript. For human primary cell culture experiments, relevant donor / patient information was fully anonymized and not visible to the researchers. For human bronchoalveolar lavage sample analysis shown in Fig. 1I, this information was available and is provided in Supplementary Table 1. As visible in this table, the distribution of male and female patients in the cohort was 50:50 (%). No sex-specific analysis was performed, as the sample size after splitting the cohort according to sex and after age-matching in this case was considered too small to yield statistically meaningful results.
Reporting on race, ethnicity, or other socially relevant groupings	No analysis focusing on race, ethnicity, or other socially relevant groupings were conducted in this study.
Population characteristics	The patient cohort for bronchoalveolar lavage cell pellet analysis had a male:female sex-distribution of 50:50 (%) and a median age of 64. Age and sex of human patients are reported in Supplementary Table 1. Indication for bronchoalveolar lavage is specified for each patient in Supplementaty Table 1.
Recruitment	Bronchoalveolar lavage (BAL) fluid (BALF) samples were collected from patients who underwent BAL for pulmonary disease assessment at the University Clinics Giessen and Marburg (various indications, specified in Supplementary Table 1). Each person, undergoing bronchoscopy including bronchoalveolar lavage (BAL), gave both oral and written informed consent. Relevant patient parameters (sex, age, indication) were only disclosed to the researchers after the RT-qPCR data hown in Fig. 1I had been recorded, to prevent from any potential bias during sample analysis.
Ethics oversight	The study was approved by the local ethics comittees of the medical faculties at Philipps University Marburg and Justus Liebig University Giessen.

Note that full information on the approval of the study protocol must also be provided in the manuscript.

Field-specific reporting

Please select the one below that is the best fit for your research. If you are not sure, read the appropriate sections before making your selection.

☒ Life sciences ☐ Behavioural & social sciences ☐ Ecological, evolutionary & environmental sciences

For a reference copy of the document with all sections, see nature.com/documents/nr-reporting-summary-flat.pdf

Life sciences study design

All studies must disclose on these points even when the disclosure is negative.

Sample size	No formal sample size calculation was performed. Instead, sample sizes were determined based on commonly accepted standards in the field and practical feasibility given the experimental setup. For RNA-seq and proteomics experiments, we used at least triplicates (three independent experiments), where possible, which is standard practice to ensure statistical power. Similarly, for qPCR validations and further functional assays, we included at least three independent experiments, with additional replicates in key experiments to strengthen reliability. Furthermore, our conclusions are supported by multiple independent lines of evidence, including consistent results across different experimental approaches, such as RNA-seq, qPCR, and proteomics, and biological reproducibility, as key findings were validated across different cell models (G-MΦ, M-MΦ, aMΦ). Stringent statistical analyses, with appropriate significance thresholds were applied to all datasets, as specified in the manuscript. Given these factors, we believe that the selected sample sizes are sufficient to support the robustness and reproducibility of our findings.
Data exclusions	Beyond standard filtering steps to exclude background noise (RPKM ≥ 0.5 threshold in RNA-seq datasets), data were only excluded if an experiment clearly failed, as determined by the assessment of positive controls. For example, in lncRNA knockdown experiments, samples were excluded if RT-qPCR validation showed no detectable reduction in the target lncRNA levels. All exclusions were based on predefined quality control criteria to ensure the integrity and reliability of the dataset.
Replication	The number of experimental replicates is indicated in the respective figure legends. If not stated differently, at least three independent experiments were performed.
Randomization	For cell culture experiments, randomization was not applicable, as experimental conditions were systematically assigned based on predefined treatment groups (e.g., LPS stimulation, knockdown conditions) rather than random allocation. For cell-based experiments, all samples were handled under identical conditions, minimizing technical variability. For patient-derived BAL samples, we analyzed variations in gene expression correlating with IFNB1 Levels and controlled for covariates by using standardized RNA input amounts and control RNA detections.

Patient information (i.e. sex, age, disease) was not disclosed to researchers prior to sample analysis.

Blinding: For BAL sample analysis, patient information (i.e. sex, age, disease) was disclosed to the researchers by the responsible clinician only retrospectively, after all samples had been analysed by RT-qPCR.

Reporting for specific materials, systems and methods

We require information from authors about some types of materials, experimental systems and methods used in many studies. Here, indicate whether each material, system or method listed is relevant to your study. If you are not sure if a list item applies to your research, read the appropriate section before selecting a response.

Materials & experimental systems

n/a	Involved in the study
☐	☒ Antibodies
☐	☒ Eukaryotic cell lines
☒	☐ Palaeontology and archaeology
☒	☐ Animals and other organisms
☒	☐ Clinical data
☒	☐ Dual use research of concern
☒	☐ Plants

Methods

n/a	Involved in the study
☒	☐ ChIP-seq
☒	☐ Flow cytometry
☒	☐ MRI-based neuroimaging

Antibodies

Antibodies used: Anti-hnRNPL antibody, mouse monoclonal (clone 4D11, Sigma-Aldrich, # R4903); Monoclonal anti-FLAG M2 antibody produced in mouse (Sigma-Aldrich, F1804); Anti-mouse-HRP, Goat IgG (Santa Cruz, sc-2005). For CoIPs, 2.5 µg of anti-hnRNPL or anti-FLAG antibody were used per capture. For Western blot analysis, anti-hnRNPL was used at a 1:10000 dilution and anti-mouse-HRP at 1:1000.

Validation: Anti-hnRNP L antibody and anti-FLAG antibody were used for Co-IPs in the present study. Specificity was validated by protein mass-spectrometry analysis of the CoIP-eluates (Fig. 6E)

Eukaryotic cell lines

Policy information about cell lines and Sex and Gender in Research

Cell line source(s): THP-1 cell were obtained from ATCC (# tib-202). Primary cells for macrophage differentiation were obtained via the Transfusion Medicine department, University Clinics Giessen and Marburg (sex of the donors not disclosed); BAL-samples from human participants were obtained via bronchoalveolar lavage (University Clinics Giessen and Marburg, sex desclosed in Table 1).

Authentication: An autenthicated THP1 cell line sample was directly purchased from ATCC and regularly inspected by microscopy and RNA-seq analysis, to validate its monocytic nature.

Mycoplasma contamination: THP1 cells, used in the present study were regularly tested for mycoplasma coontamination. All test results were negative.

Commonly misidentified lines (See ICLAC register): None.

Plants

Seed stocks: *Report on the source of all seed stocks or other plant material used. If applicable, state the seed stock centre and catalogue number. If plant specimens were collected from the field, describe the collection location, date and sampling procedures.*

Novel plant genotypes: *Describe the methods by which all novel plant genotypes were produced. This includes those generated by transgenic approaches, gene editing, chemical/radiation-based mutagenesis and hybridization. For transgenic lines, describe the transformation method, the number of independent lines analyzed and the generation upon which experiments were performed. For gene-edited lines, describe the editor used, the endogenous sequence targeted for editing, the targeting guide RNA sequence (if applicable) and how the editor was applied.*

Authentication: *Describe any authentication procedures for each seed stock used or novel genotype generated. Describe any experiments used to assess the effect of a mutation and, where applicable, how potential secondary effects (e.g. second site T-DNA insertions, mosiacism, off-target gene editing) were examined.*
